# Biological role of EPS from *Pseudomonas syringae* pv. syringae UMAF0158 extracellular matrix, focusing on a Psl-like polysaccharide

**DOI:** 10.1038/s41522-020-00148-6

**Published:** 2020-10-12

**Authors:** Zaira Heredia-Ponce, Jose Antonio Gutiérrez-Barranquero, Gabriela Purtschert-Montenegro, Leo Eberl, Francisco M. Cazorla, Antonio de Vicente

**Affiliations:** 1 Instituto de Hortofruticultura Subtropical y Mediterránea “La Mayora” (IHSM-UMA-CSIC) - Departamento de Microbiología, Universidad de Málaga, Bulevar Louis Pasteur, 31 (Campus Universitario de Teatinos), 29071 Málaga, Spain; 2 Department of Plant and Microbial Biology, University of Zurich. Zollikerstrasse 107, CH-8008 Zurich, Switzerland

**Keywords:** Biofilms, Pathogens

## Abstract

*Pseudomonas syringae* is a phytopathogenic model bacterium that is used worldwide to study plant–bacteria interactions and biofilm formation in association with a plant host. Within this species, the syringae pathovar is the most studied due to its wide host range, affecting both, woody and herbaceous plants. In particular, *Pseudomonas syringae* pv. syringae (Pss) has been previously described as the causal agent of bacterial apical necrosis on mango trees. Pss exhibits major epiphytic traits and virulence factors that improve its epiphytic survival and pathogenicity in mango trees. The cellulose exopolysaccharide has been described as a key component in the development of the biofilm lifestyle of the *P. syringae* pv. syringae UMAF0158 strain (PssUMAF0158). PssUMAF0158 contains two additional genomic regions that putatively encode for exopolysaccharides such as alginate and a Psl-like polysaccharide. To date, the Psl polysaccharide has only been studied in *Pseudomonas aeruginosa*, in which it plays an important role during biofilm development. However, its function in plant-associated bacteria is still unknown. To understand how these exopolysaccharides contribute to the biofilm matrix of PssUMAF0158, knockout mutants of genes encoding these putative exopolysaccharides were constructed. Flow-cell chamber experiments revealed that cellulose and the Psl-like polysaccharide constitute a basic scaffold for biofilm architecture in this bacterium. Curiously, the Psl-like polysaccharide of PssUMAF0158 plays a role in virulence similar to what has been described for cellulose. Finally, the impaired swarming motility of the Psl-like exopolysaccharide mutant suggests that this exopolysaccharide may play a role in the motility of PssUMAF0158 over the mango plant surface.

## Introduction

*Pseudomonas syringae* is a model bacterium for the study of plant–microbial interactions, as it causes diseases in woody and herbaceous plants worldwide. Mainly based on host isolation and host range, *P. syringae* is divided into more than 60 pathovars^1^, among which pathovar syringae shows the largest host range, causing disease in over 180 plant species^2^. *P. syringae* shows two interconnected lifestyles while interacting with the plant: an epiphytic phase, in which it survives on the surface while coping with harsh environmental conditions, and a pathogenic phase, in which it enters and colonizes internal plant tissue, leading to the development of an infection^2–4^. The *P. syringae* pv. syringae (Pss) UMAF0158 strain (PssUMAF0158) is a mango tree pathogen that is considered a model for the study of the transition between the epiphytic and pathogenic lifestyles depending on environmental conditions^5^.

*P. syringae* harbours a diverse weaponry of virulence factors, including the type III secretion system (T3SS) and its effectors, phytotoxins, phytohormones, ice nucleation activity, plant cell wall-degrading enzymes and exopolysaccharides^3^. The ability to produce exopolysaccharides has been previously related to virulence in several phytopathogenic bacteria^6–9^. *P. syringae* produces a number of biofilm matrix polysaccharides, including alginate, levan and cellulose^9–14^. Alginate is a copolymer of O-acetylated β-1,4-linked D-mannuronic acid and L-glucuronic acid that has been widely studied in *P. syringae*^11,12,15,16^ and *Pseudomonas aeruginosa*^17–19^. Generally, the role of alginate during biofilm formation in these two species has been considered nonessential^16–18^. However, several studies have shown that alginate plays a role in the epiphytic fitness and virulence in some *P. syringae* strains^7,20^, as well as in biofilm structure, antibiotic resistance and protection against the human immune system in mucoid strains of *P. aeruginosa*^19,21,22^. The polysaccharide levan is a β-2,6 polyfructan that shows extensive branching through β-2,1 linkages^16^ whose synthesis is catalysed by levansucrases^10,13^. Levan does not play a role in biofilm architecture, and it has been speculated to consist of a storage molecule that may protects cells against starvation^16^. Cellulose is a polymer composed of β-D-glucose units that constitutes one of the main components of the biofilm matrix produced by many bacteria^23–26^, and its biosynthesis has proven to be important for biofilm formation by Pss^9,27^.

The PssUMAF0158 genome sequencing project revealed the presence of a gene cluster related to cellulose biosynthesis^28^. This gene cluster was identified as being closely related to the lifestyle of PssUMAF0158 on the mango tree surface^9^. Cellulose overexpression reduces virulence, whereas cellulose-deficient mutants increase the area of necrosis^9^. This suggests that cellulose could act as a switch in the transition between epiphytic and pathogenic phases, decreasing cellulose biosynthesis and thus, biofilm formation, in the pathogenic phase^4^. In addition to alginate and cellulose, a region that putatively encodes a Psl-like exopolysaccharide was found in the PssUMAF0158 genome in this study. The Psl polysaccharide, composed of D-mannose, D-glucose and L-rhamnose^29^, has thus far only been studied in *P. aeruginosa*^30–32^, where it plays essential roles in biofilm formation, adhesion, motility and protection against a variety of stresses^33–38^. Although the presence of the Psl polysaccharide has been reported in a few species of the *Pseudomonas* genus, including the plant-associated *P. syringae* pv. syringae B728a and *P. syringae* pv. phaseolicola 1448a strains, the putative roles that this polysaccharide could play in biofilm formation in these bacteria have not been examined yet^39,40^.

Biofilm formation could play an important role in the PssUMAF0158 lifestyle during its interaction with the mango tree surface, so further research regarding its biofilm components and how they establish interactions with each other to promote epiphytic survival is needed. In this study, in addition to cellulose, whose roles in biofilm formation and virulence have been previously reported, we have identified two genomic regions that putatively encode alginate and Psl-like exopolysaccharides. Thus, the main aim of this work is to elucidate the roles that these exopolysaccharides play in biofilm formation and architecture, as well as virulence, during interaction with the mango plant.

## Results

### Bioinformatic analysis revealed that alginate- and Psl-like exopolysaccharides encoding clusters were present in the *Pseudomonas syringae* pv. syringae UMAF0158 genome

The presence of the alginate and *psl*-like gene clusters has never been assessed in PssUMAF0158 strain. An in silico analysis was performed to identify the genome regions that may be encoding these exopolysaccharides in PssUMAF0158. Using the alginate operon sequence of *P. syringae* pv. syringae B728a as a model, the Psyrmg_RS21275-Psyrmg_RS21330 region was identified in PssUMAF0158 (Supplementary Fig. 1). There is high conservation pattern between the proteins encoded by these regions. The Psyrmg_RS06720-Psyrmg_RS06770 genomic region of PssUMAF0158 has been found to be similar to the *psl* operon of *P. aeruginosa* PAO1, although with some differences (Supplementary Fig. 2). The PslM-like and PslO-like proteins were missing, although they are not required to produce the polysaccharide^29^. PslC-like and PslN-like proteins seemed to be encoded somewhere else on the chromosome at Psyrmg_RS00890 and Psyrmg_RS04445 (Supplementary Table 1b). The PslL acyltransferase of PAO1 shares no identity with any protein encoded by the genome of PssUMAF0158. However, the Psyrmg_RS06765 gene, located within the *psl*-like cluster, encodes for an acetyltransferase. The identity between the proteins was over fifty percent (Supplementary Table 1b) and most of the domains were conserved (Supplementary Table 1a, b). The cellulose operon of *P. syringae* pv. tomato DC3000 (PtDC3000) was previously reported to be orthologous to the Psyrmg_RS20465-Psyrmg_RS20505 region in PssUMAF0158^9^ (Supplementary Fig. 3). There is high conservation pattern between the proteins encoded by these cellulose production *loci*.

### Phylogenetic analysis revealed that a *psl*-like gene cluster was present on strains of the *Pseudomonas syringae* complex that interact with plants

**Fig. 1:**
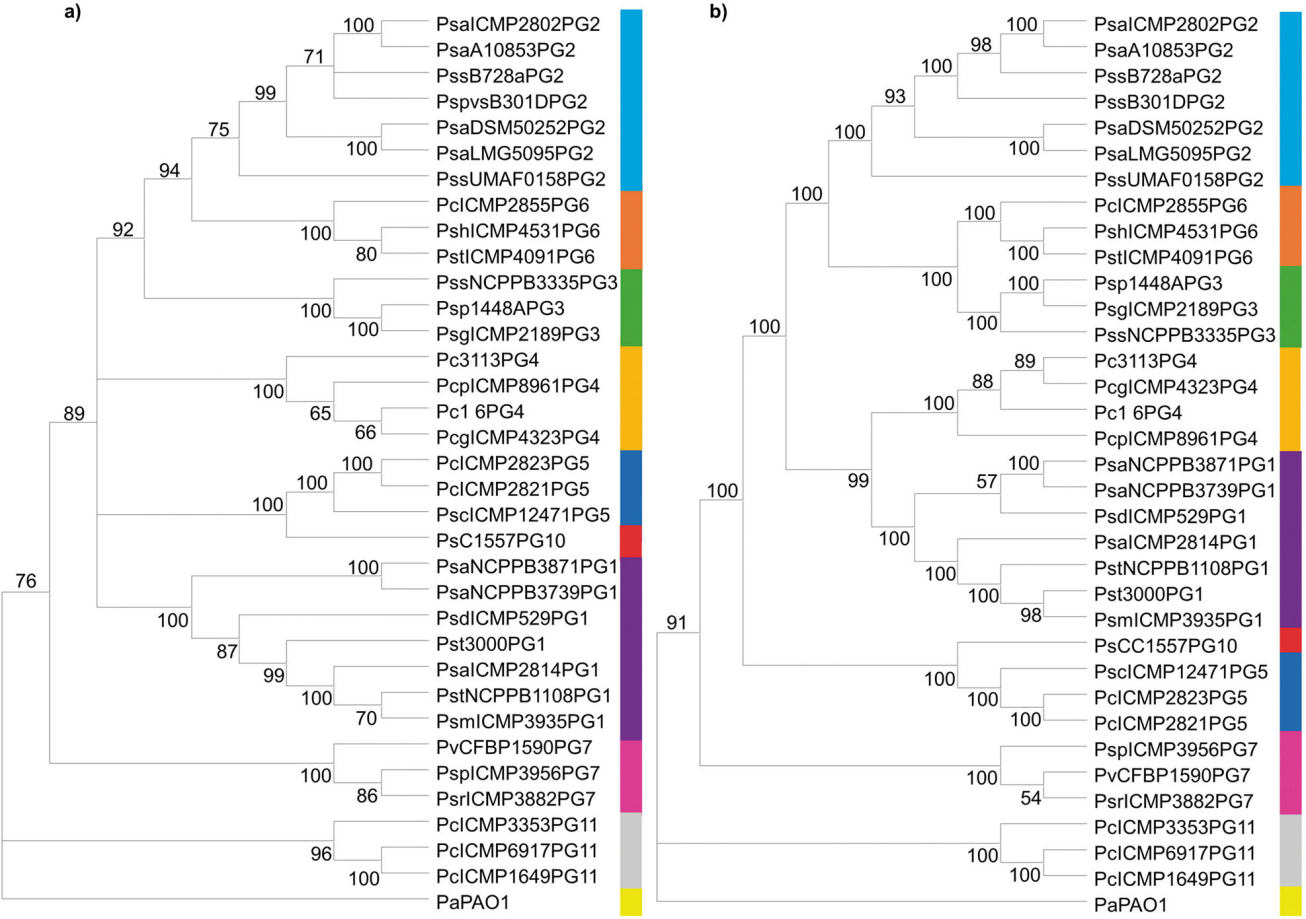
Evolutionary history of the Psl-like exopolysaccharide genomic cluster in plant-associated phylogroups of the *P. syringae* complex. **a** Neighbour-joining tree generated with MEGA10 using partial combined sequences of the *rpoD* and *gyrB* genes. **b** Neighbour-joining tree generated with MEGA10 using the *psl-*like cluster nucleotide sequence. Both analyses included 34 strains belonging to 1 (purple), 2 (light blue), 3 (green), 4 (dark yellow), 5 (dark blue), 6 (orange), 7 (pink), 10 (red) and 11 (grey) phylogenetic groups within the *P. syringae* complex (Supplementary Table 2). The *P. aeruginosa* PAO1 *psl* operon sequence was used as an outgroup (light yellow).

To elucidate the evolutionary history of the *psl*-like gene cluster within the *Pseudomonas syringae* complex, a total of 34 strains belonging to phylogenetic groups 1, 2, 3, 4, 5, 6, 7, 10 and 11, mainly related to plants, were selected and used for the analysis (Supplementary Table 2). The partial sequences of the *rpoD* and *gyrB* housekeeping genes clearly supported the reported phylogenetic distribution in the different phylogenetic subgroups included in the analysis^41^ (Fig. 1a). Therefore, the phylogenetic distribution of the strains from the different phylogenetic groups regarding the *psl*-like gene cluster indicated that this cluster followed a similar evolutionary history to that of the housekeeping genes and demonstrated that it has been stably and vertically inherited by this group of microorganisms (Fig. 1b).

### Involvement of the Psl-like exopolysaccharide in the virulence and plant adhesion of *P. syringae* pv. syringae UMAF0158

**Fig. 2:**
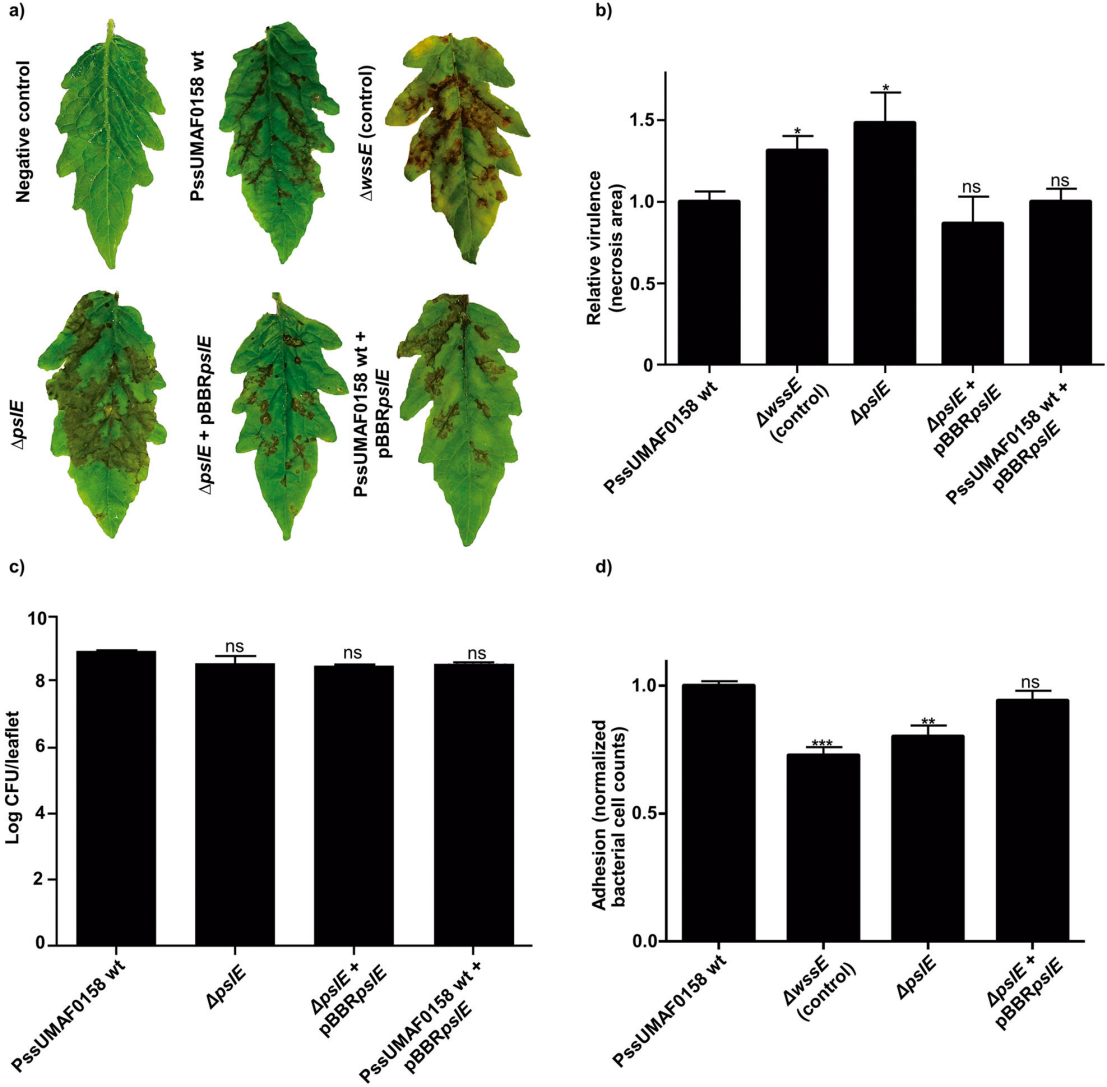
Analysis of the Psl-like polysaccharide as a putative virulence factor and adhesion factor for *P. syringae* pv. syringae UMAF0158. Virulence determination on inoculated tomato leaflets maintained in vitro. **a** Representative symptoms developed on tomato leaflets at 6 days postinoculation. **b** Relative virulence of PssUMAF0158 wild-type and Psl-like polysaccharide mutant in tomato leaflets measured by lesion size. The cellulose mutant was included as a positive virulence control^9^. Four leaflets per experiment, and three independent experiments were performed. **c** Bacterial counts (log CFU/ml) after 6 days of inoculation. **d** Adhesion to mango leaves at 4 h postinoculation. The cellulose mutant was included as a negative adhesion control^9^. Normalized bacterial cell counts recovered from mango leaves of the different assayed mutants with respect to the wild-type strain counts. The PssUMAF0158 wild-type (PssUMAF0158 wt), PssUMAF0158 cellulose mutant (*ΔwssE*), PssUMAF0158 Psl-like polysaccharide mutant (*ΔpslE*), PssUMAF0158 Psl-like complemented strain (*ΔpslE* + pBBR*pslE*) and PssUMAF0158 *pslE* overexpressing strain (PssUMAF0158 wt+pBBR*pslE*) were tested. Statistical significance was assessed by two-tailed Mann–Whitney test (*p < 0.05, **p < 0.01, ***p < 0.001). Error bars correspond to the standard error of the mean (s.e.m.).

Virulence experiments were performed on tomato leaflets, which are a more reliable plant model for pathogenicity than mango leaves^9,42^. At day six postinoculation, the overall necrotic area was estimated, and the results demonstrated significant differences between PssUMAF0158 wild-type and mutant defective in Psl-like exopolysaccharide production (Fig. 2a, b). The cellulose mutant was included as a positive control of virulence^9^. No significant differences in virulence were found between the wild-type and the *Δalg8* mutant (Supplementary Fig. 4). Moreover, the bacterial counts were similar between the tested strains (Fig. 2c), which indicates that the greater virulence observed in the *ΔpslE* mutant was not due to an increase in the ability to grow on the leaflet surface. In addition, adhesion experiments were performed on mango leaves using the mutant defective in Psl-like exopolysaccharide production (Fig. 2d). The cellulose mutant was included as an impaired control of adhesion^9^. The results showed a significant reduction in adhesion to mango leaves in the mutant compared to the wild-type and demonstrated that the Psl-like complemented strain restored adhesion to the wild-type levels (Fig. 2d).

### Effects of *alg8*, *wssE* and *pslE* mutations on colony morphology and Congo red binding

**Fig. 3:**
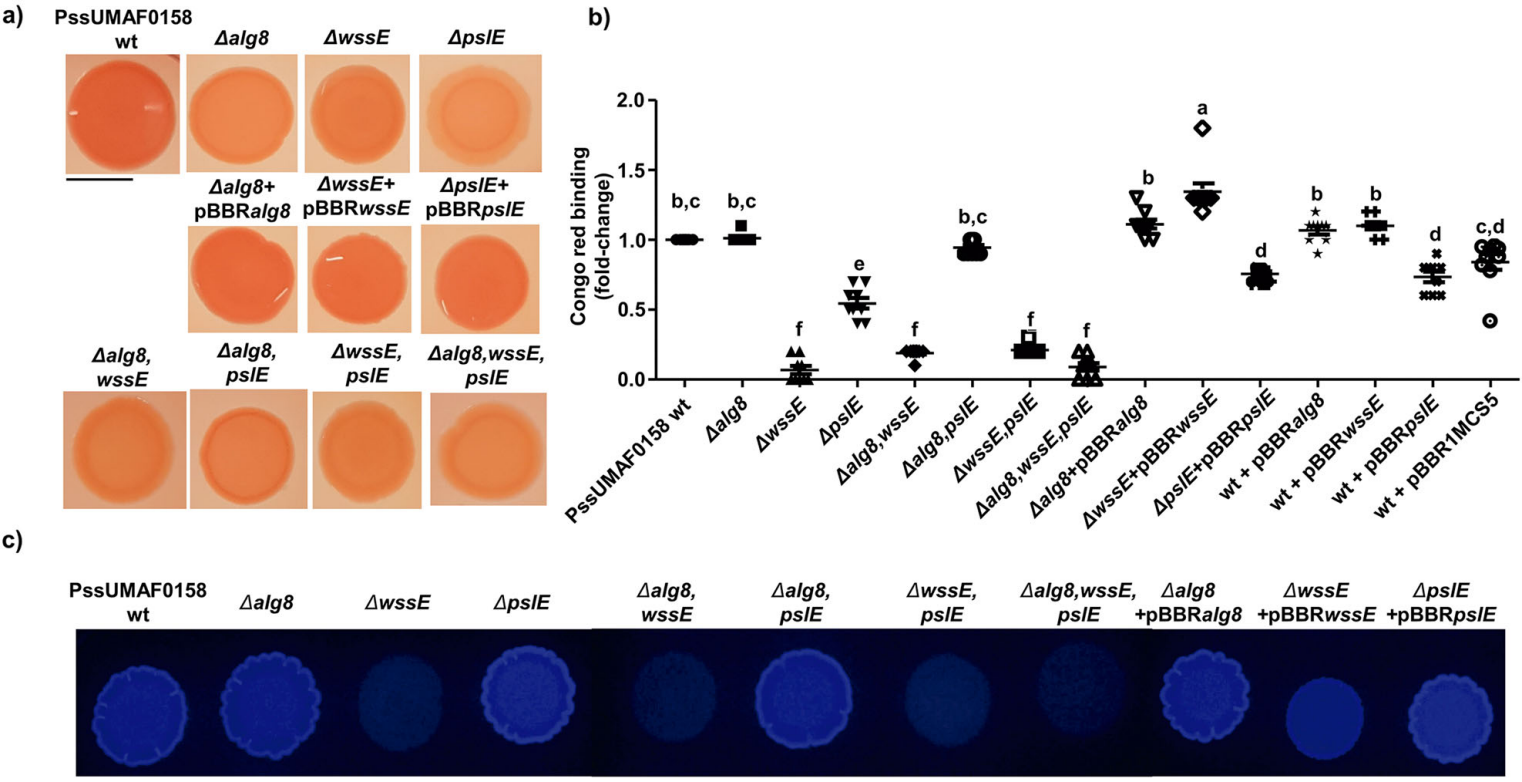
Congo red binding and colony morphology. **a** Plate CR binding assay and colony morphology of wild-type, mutants and complemented strains. **b** Pellicle CR binding assay. The results show the CR binding levels of the pellicle in the form of a fold-change relative to the wild-type strain CR binding average. **c** Plate calcofluor binding assay. The PssUMAF0158 wild-type (PssUMAF0158 wt), PssUMAF0158 alginate mutant (*Δalg8*), PssUMAF0158 cellulose mutant (*ΔwssE*), PssUMAF0158 Psl-like polysaccharide mutant (*ΔpslE*), PssUMAF0158 *Δalg8,wssE* double mutant (*Δalg8,wssE*), PssUMAF0158 *Δalg8,pslE* double mutant (*Δalg8,pslE*), PssUMAF0158 *ΔwssE,pslE* double mutant (*ΔwssE,pslE*), PssUMAF0158 *Δalg8,wssE,pslE* triple mutant (*Δalg8,wssE,pslE*), alginate complemented strain (*Δalg8* + pBBR*alg8*), cellulose complemented strain (*ΔwssE* + pBBR*wssE*), Psl-like complemented strain (*ΔpslE* + pBBR*pslE*), *alg8* overexpression strain (wt + pBBR*alg8*), *wssE* overexpression strain (wt + pBBR*wssE*), *pslE* overexpression strain (wt + pBBR*pslE*) and vector control strain (wt + pBBR1MCS5) were tested. Statistical analysis was performed using ANOVA with the Bonferroni correction test. Three replicates, and three independent experiments were performed. Different letters represent statistically significant differences, *p* < 0.05. Error bars show the standard error of the mean (s.e.m.). Scale bar 1 cm.

The Congo red (CR) binding observed in colonies of wild-type and derivative mutants suggests that these genes are involved in the production of exopolysaccharides. CR agar plates showed that PssUMAF0158 wild-type colonies were dark red, while colonies of the mutants were pale pink (Fig. 3a). Complemented strains restored the wild-type phenotype, and no differences in colony morphology were observed. The pellicle CR binding experiments (Fig. 3b) showed some differences with the plate CR binding experiments (Fig. 3a). The *Δalg8,pslE* double mutant, which was impaired in plate CR binding compared to the wild-type (Fig. 3a), restored to the wild-type levels in pellicle CR binding (Fig. 3b). Increases of cellulose production were not observed in the *Δalg8,pslE* strain in the calcofluor staining experiments (Fig. 3c). Overexpression of the *wssE* gene was also not detected at 4-, 6- and 16 h postinoculation (Supplementary Fig. 6). There were also differences between plate CR binding and pellicle CR binding in the alginate mutant. Cellulose mutant strain complemented with the *wssE* gene present on the pBBR1MCS5 plasmid fully restored to the wild-type phenotype, but the Psl-like mutant strain complemented with the *pslE* gene present on the same plasmid only partially restored CR binding in the pellicle. As observed in the CR binding phenotype of the pellicles of the vector control strain (wt + pBBR1MCS5), the plasmid is not affecting CR binding under these conditions. The decrease in CR binding observed in the wt + pBBR*pslE* strain suggests that expression of the *pslE* gene under the P_lac_ promoter present on the pBBR1MCS5 plasmid could affect CR binding in the pellicle. The *wssE* gene deletion completely impaired the ability to bind CR in the pellicle (Fig. 3b). The pellicle CR binding phenotypes observed (Fig. 3b) match with the ability of the tested strains to produce cellulose (Fig. 3c).

### The genes involved in the production of the cellulose and Psl-like polysaccharides are essential for biofilm formation

**Fig. 4:**
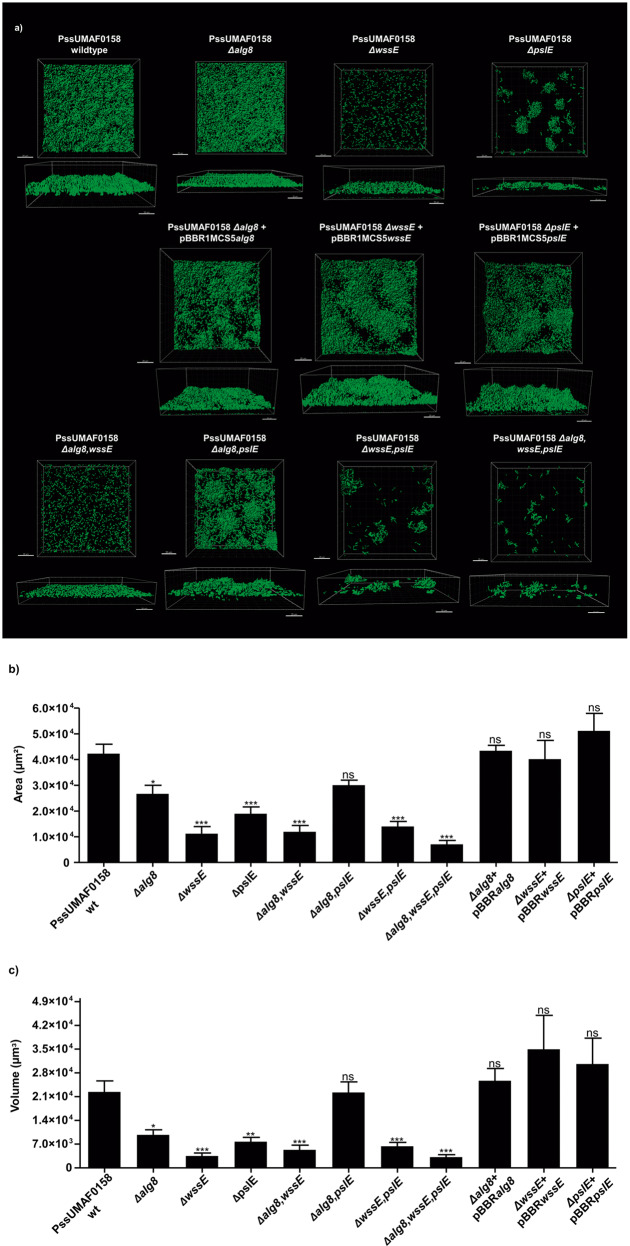
Flow-cell chamber experiments of PssUMAF0158 wild-type and derived extracellular matrix mutants. **a** Representative 48 h 3D biofilm images of GFP-tagged PssUMAF0158 wild-type and mutants are shown. The obtained images were analysed with the Leica Application Suite (Mannheim, Germany) and the IMARIS software package (Bitplane, Switzerland). Scale bar 20 µm. **b** Area in the field of view covered by 48 h biofilms of the GFP-tagged PssUMAF0158 wild-type, extracellular matrix mutants and complemented strains. **c** Volume in the field of view occupied by 48 h biofilms of the GFP-tagged PssUMAF0158 wild-type, extracellular matrix mutants and complemented strains. The area and volume values were calculated with the IMARIS software package (Bitplane, Switzerland). The following GFP-tagged strains were tested: PssUMAF0158 wild-type (PssUMAF0158 wt), PssUMAF0158 alginate mutant (*Δalg8*), PssUMAF0158 cellulose mutant (*ΔwssE*), PssUMAF0158 Psl-like polysaccharide mutant (*ΔpslE*), PssUMAF0158 *Δalg8,wssE* double mutant (*Δalg8,wssE*), PssUMAF0158 *Δalg8,pslE* double mutant (*Δalg8,pslE*), PssUMAF0158 *ΔwssE,pslE* double mutant (*ΔwssE,pslE*), PssUMAF0158 *Δalg8,wssE,pslE* triple mutant (*Δalg8,wssE,pslE*), alginate complemented strain (*Δalg8* + pBBR*alg8*), cellulose complemented strain (*ΔwssE* + pBBR*wssE*) and Psl-like complemented strain (*ΔpslE* + pBBR*pslE*). A minimal of three replicates, and three independent experiments were performed. Statistical significance was assessed by two-tailed Mann–Whitney test (*p < 0.05, **p < 0.01, ***p < 0.001). Error bars show the standard error of the mean (s.e.m.).

To investigate the role of alginate, cellulose and Psl-like exopolysaccharides in the biofilm architecture, flow-cell chamber experiments were performed in the wild-type and mutant bacteria, and confocal laser scanning microscopy (CLSM) was used to visualize live biofilms. A group of cells that were tightly joined together and motionless over the flow-cell chamber surface was considered a cell aggregate, as previously illustrated^34^. Area and volume values in the field of view were calculated to evaluate the surface coverage and the overall biofilm architecture of each strain, respectively. After 48 h, the wild-type PssUMAF0158 formed thick biofilms with cell aggregates (Fig. 4). The *Δalg8* mutant exhibited a significantly lower surface coverage (Fig. 4b) and the overall biofilm architecture appeared to be flattened compared to that of the wild-type strain (Fig. 4c). The cellulose mutant showed an impairment in biofilm formation, characterized by the absence of cell aggregates (Fig. 4). The PssUMAF0158 *ΔpslE* mutant produced a substantially altered biofilm characterized by scattered cell aggregates across the surface (Fig. 4). As observed previously in the CR binding experiments of the pellicles, the biofilms formed by the *Δalg8,pslE* double mutant restored the wild-type phenotype in area and volume values. The PssUMAF0158 *Δalg8,wssE,pslE* triple mutant was almost completely impaired in biofilm formation (Supplementary Fig. 7).

### Cellulose is a component of PssUMAF0158 biofilms

To observe the presence of cellulose polysaccharide in PssUMAF0158 biofilms, calcofluor staining was performed using both flow-cell chambers (Supplementary Fig. 5) and plate assays (Fig. 3c). As expected, cellulose staining was absent in the *ΔwssE* mutant. Flow-cell chamber experiments allowed us to observe that cellulose is located in the cell aggregates of the PssUMAF0158 wild-type strain (Supplemental Fig. 5). In *trans* expression of the *wssE* gene restored cellulose mutant phenotype to the wild-type levels (Fig. 3c).

**Fig. 5:**
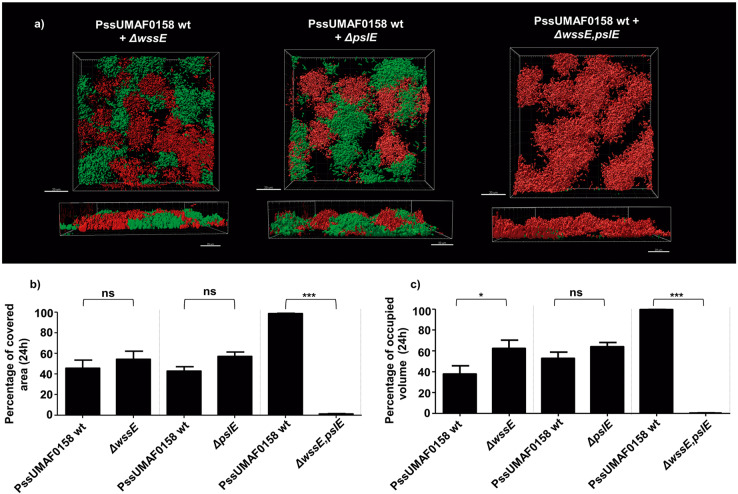
Competition in mixed biofilms. Role of different polysaccharides in competition for biofilm formation. **a** Representative 24 h 3D images of mixed biofilms including the dsRed-tagged PssUMAF0158 wild-type and GFP-tagged matrix mutants. The obtained images were analysed with the Leica Application Suite (Mannheim, Germany) and the IMARIS software package (Bitplane, Switzerland). Scale bar 20 µm. **b** Percentage of the area occupied by the wild-type and the respective mutants after 24 h of competition calculated with IMARIS software. **c** Percentage of the volume occupied by the wild-type and the respective mutants after 24 h of competition calculated with IMARIS software. The dsRed-tagged PssUMAF0158 wild-type (PssUMAF0158 wt), GFP-tagged PssUMAF0158 cellulose mutant (*ΔwssE*), GFP-tagged PssUMAF0158 Psl-like polysaccharide mutant (*ΔpslE*) and GFP-tagged PssUMAF0158 *ΔwssE,pslE* double mutant (*ΔwssE,pslE*) were tested. A minimal of two replicates and three independent experiments were performed. Statistical significance was assessed by two-tailed Mann–Whitney test (*p < 0.05, **p < 0.01, ***p < 0.001). Error bars show the standard error of the mean (s.e.m.).

### Both cellulose and Psl-like exopolysaccharides are necessary for the competition of *P. syringae* pv. syringae UMAF0158 in biofilm formation

To investigate the roles of the main exopolysaccharides implicated in biofilm formation in bacterial competition and niche colonization, mixed biofilms containing the dsRed-tagged wild-type and GFP-tagged mutants for cellulose and/or Psl-like gene clusters were assessed in flow-cell chambers (Fig. 5). When just one of the two most relevant polysaccharides were missing, each strain occupied around fifty percent of the colonized space, which indicates that there was no impairment in niche colonization by the mutants compared to the wild-type strain (Fig. 5b, c). However, the double mutant was not able to compete with the wild-type, since it occupied about two percent of the colonized space compared to the nighty-eight percent of the wild-type strain. This result suggests a synergistic role of the two polysaccharides during colonization.

### The Psl-like polysaccharide plays a role in swarming motility

**Fig. 6:**
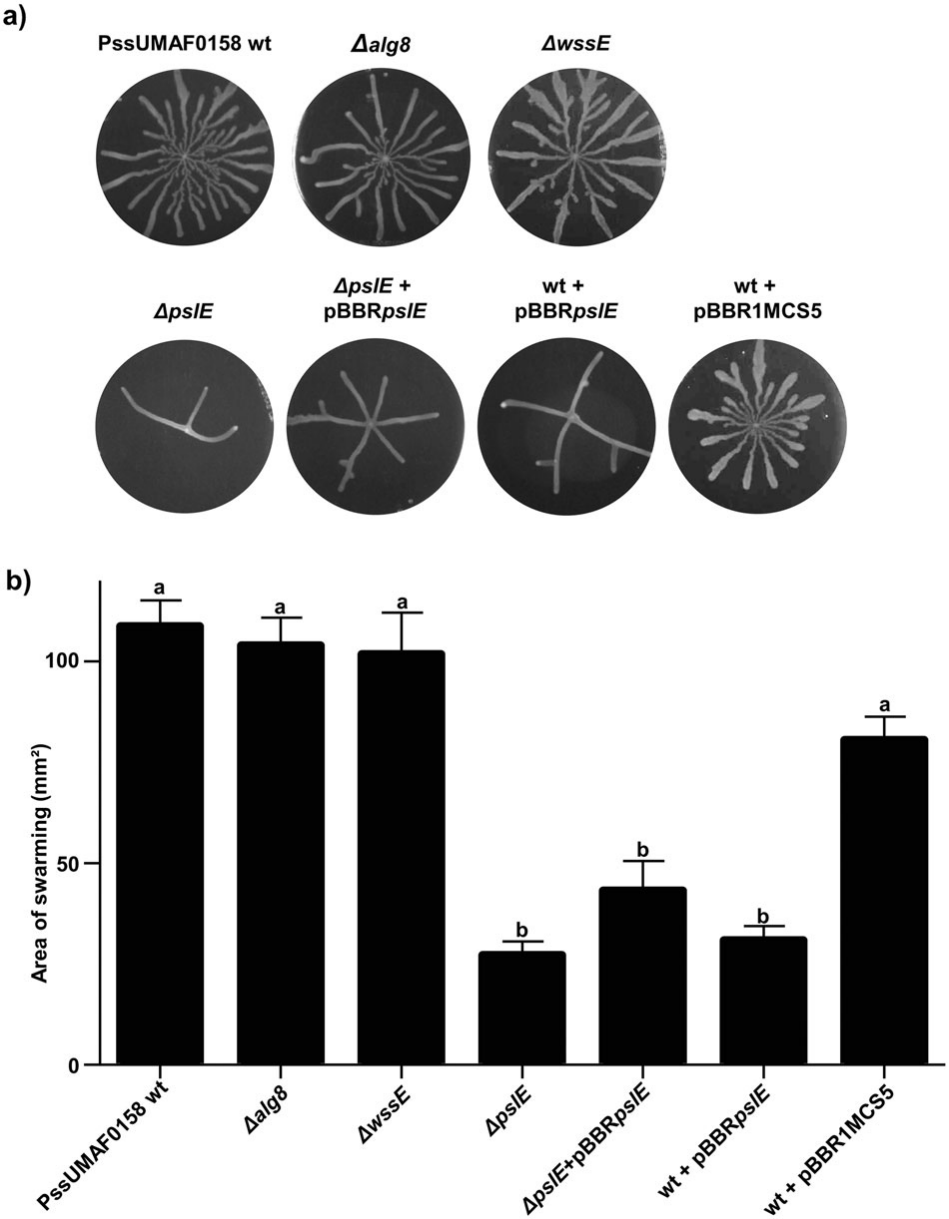
Swarming motility. Effect of polysaccharide production on swarming motility. **a** Representative images of swarming plates incubated at 25 °C at 48 h postinoculation. **b** Swarm motility area after 48 h of growth at 25 °C. The PssUMAF0158 wild-type (PssUMAF0158 wt), PssUMAF0158 alginate mutant (*Δalg8*), PssUMAF0158 cellulose mutant (*ΔwssE*), PssUMAF0158 Psl-like polysaccharide mutant (*ΔpslE*), Psl-like complemented strain (*ΔpslE* + pBBR*pslE*), *pslE* overexpression strain (wt + pBBR*pslE*) and vector control (wt + pBBR1MCS5) were tested. Three plates per experiment, and three independent experiments were performed. Statistical analysis was performed using ANOVA with the Bonferroni correction test. Three replicates, and three independent experiments were performed. Different letters represent statistically significant differences, *p* < 0.05. Error bars show the standard error of the mean (s.e.m.).

Swarming experiments were performed using the PssUMAF0158 wild-type and extracellular matrix mutants. Swarming patterns occurred as migrating and branching tendrils from the point of inoculation. Among all the extracellular matrix mutants included in this study, only PssUMAF0158 *ΔpslE* mutant, and the double and triple mutants that included the *pslE* gene deletion (Supplementary Fig. 8), were impaired in swarming motility (Fig. 6). The Psl-like complemented strain did not exhibit significant restoration of the wild-type swarming motility phenotype in these conditions. Analysis of transcript abundance of the *pslD* and *pslF* genes in the wild-type and *ΔpslE* mutant strains (Supplementary Fig. 9) revealed differences in gene expression under the analysed conditions. Furthermore, *pslE* expression under the P_lac_ promoter present on the pBBR1MCS5 plasmid could also affect swarming in these conditions, as it was observed in the swarming phenotype of PssUMAF0158 wt + pBBR*pslE* control strain. These facts could explain why swarming motility did not restore to the wild-type phenotype in the Psl-like complemented strain.

## Discussion

Biofilms play an important role in the lifestyle of the phytopathogenic bacterium *P. syringae* pv. syringae UMAF0158 (PssUMAF0158), particularly on mango tree surfaces^4,9^. Beyond cellulose production by PssUMAF0158, little is known about the composition of PssUMAF0158 extracellular matrix. Therefore, we investigated the biological roles of two gene clusters other than those related to cellulose production that seem to be involved in the synthesis of alginate and a Psl-like exopolysaccharides in PssUMAF0158. The roles that Psl polysaccharide plays in non-*aeruginosa Pseudomonas* remain unknown. Then, it is noteworthy that the *psl*-like cluster was found in all the main plant-associated phylogenetic groups included in the *P. syringae* complex (Fig. 1). The phylogenetically maintained *psl*-like cluster suggests that this polysaccharide could be relevant not only for PssUMAF0158 lifestyle, but also among all the plant-associated phylogroups of the *P. syringae* complex. Taking this into account, we mainly concentrated our efforts on discerning the function of the *psl*-like gene cluster in PssUMAF0158.

Pss transitions between an epiphytic and a pathogenic lifestyle on mango surfaces^3–5^. Cellulose has proven to be an important component of the extracellular matrix that influences this transition, as the PssUMAF0158 cellulose-defective mutants are more virulent than the wild-type strain, and virulence is practically abolished in the cellulose-overproducing strain^9^. Thus, biofilm formation, through cellulose biosynthesis, could be favoured in the epiphytic phase, and transition to the pathogenic phase could be promoted by a reduction in biofilm formation, led by a decrease in cellulose production^4^. Actually, something similar was observed in *Salmonella enterica*; when cellulose biosynthesis was repressed by MgtC, the bacteria became more virulent^43^. Psl-like polysaccharide performed a role in virulence similar to that observed for cellulose (Fig. 2a, b), which suggests that the transition between epiphytic and pathogenic lifestyles was not limited to a single component of the extracellular matrix. This redundancy might be important to rescue the epiphytic lifestyle when environmental conditions are adverse for cellulose production, and vice versa. In fact, redundant biological functions between biofilm components are not unusual^44,45^. As previously observed for cellulose^9^, adhesion experiments on mango leaves reported the influence of Psl-like polysaccharide production on the PssUMAF0158 epiphytic lifestyle (Fig. 2d). These results are consistent with other studies, in which several exopolysaccharides have been shown to play roles in cell-surface interactions^33,46,47^. Alginate biosynthesis has been previously studied in Pss^7,11,48^, but the role that it plays in the PssUMAF0158 strain, a mango tree pathogen, has not been investigated thus far. An essential role of alginate in virulence, biofilm formation or motility was not proven in this study (Figs. S4, 4 and 6, respectively). This first observation was in accord with other studies in which some alginate-defective mutants have been shown to be unaffected in the induction of symptoms^48,49^.

The extracellular matrix of Pss includes the three polysaccharides analysed in this study—alginate, cellulose and Psl-like polysaccharide, as revealed the CR and calcofluor binding experiments (Fig. 3 and Supplementary Fig. 5). The differences observed between plate CR binding (Fig. 3a) and pellicle CR binding (Fig. 3b) in the PssUMAF0158 *Δalg8* mutant suggest that this polysaccharide is more important to produce biofilms in agar plates than in broth medium. This is supported by previous works, where it was observed that alginate production in several *Pseudomonas* species, including *P. syringae*, was greater in agar plates than in broth medium^15,50^.

In contrast to what had been previously reported for alginate in *P. syringae*^16^, our results revealed a slightly contribution of this polysaccharide to the biofilm matrix of the PssUMAF0158 strain (Fig. 4). As observed in PAO1^34^, the PssUMAF0158 *Δalg8* mutant formed fewer cell aggregates than the wild-type strain in flow-cell chamber experiments (Fig. 4). The cell aggregates formed by the PssUMAF0158 *ΔpslE* mutant were disrupted in PssUMAF0158 *ΔwssE,pslE* double mutant. This is similar to the *P. aeruginosa* E2, S54485 and 19660 flow-cell chamber phenotypes, in which the *Δpsl* mutants formed small aggregates, and these aggregates were disrupted in the *Δpsl,pel* double mutants^44^. The Pel polysaccharide, which is missing in PssUMAF0158, has been described in *P. aeruginosa* as a glucose-rich exopolysaccharide, similar to cellulose^30^. These aggregates were disrupted in both species when either cellulose or Pel were not produced, which suggest they could be performing similar roles in their biofilm architectures. The fact that the cellulose mutant was unable to form cell aggregates (Fig. 4 and Supplementary Fig. 5), and that cellulose preferentially locates in them (Supplementary Fig. 5), support this suggestion. Furthermore, the restoration of the wild-type phenotype in the *Δalg8,pslE* double mutant regarding CR binding experiments of the pellicles (Fig. 3b) and biofilm area and volume values (Fig. 4b, c), suggest that another polysaccharide, such as cellulose, could be being overexpressed in PssUMAF0158 *Δalg8,pslE* double mutant the same way Pel does in PAO1 *Δalg8,pslA* double mutant^34^. Although our results lead to these suggestions, there was not noticeable cellulose overexpression in plate assays (Fig. 3a, c) or w*ssE* gene overexpression in the *Δalg8,pslE* pellicles (Supplementary Fig. 6). However, cellulose biosynthesis can also be regulated at post-translational levels^51^. Besides, our results also indicate that cellulose and Psl-like polysaccharides cooperate for niche colonization in PssUMAF0158 strain, as the *ΔwssE,pslE* double but not the single mutants were outcompeted by the wild-type when they were coinoculated in flow-cell chambers (Fig. 5). This cooperation may explain why the *psl*-gene cluster is widely conserved among pseudomonads that also produce cellulose^40^. In contrast to our findings, PAO1 *Δpsl* mutant was unable to compete for biofilm formation with PAO1 wild-type^31^.

Swarming motility is related to biofilm formation, as the two processes are frequently co-regulated^52–56^. Biosurfactants have been frequently associated with bacterial motility, since for many strains swarming motility on semi-solid agar plates is dependent upon such compounds^57–59^. In fact, in *P. aeruginosa* PAO1, the production of Psl and/or Pel polysaccharides is correlated with rhamnolipid production^37^. We decided to analyse *rhlA* expression by q-RT-PCR in the *ΔpslE* mutant, which synthetizes the rhamnolipid precursor HAA, as PssUMAF0158 strain lacks the *rhlB* and *rhlC* genes. However, swarming motility does not strictly require rhamnolipid production, as HAA itself can act as wetting agent^60^. We found downregulation of the *rhlA* gene in the *ΔpslE* mutant compared to the wild-type strain (Supplementary Fig. 10), which could explain the reduction of motility observed in the *ΔpslE* mutant (Fig. 6). However, it is interesting to point out that the relationship between biofilm formation, rhamnolipid production and motility in PssUMAF0158 seems opposite to that described in *P. aeruginosa*, as PAO1 *Δpsl* mutant showed an increase in swarming motility due to a higher rhamnolipid production^37^. The swarming impairment observed in PssUMAF0158 *ΔpslE* mutant suggests a potential role of this polysaccharide in the movement of PssUMAF0158 over the plant surface. In fact, the Psl exopolysaccharide is also involved in surface colonization in *P. aeruginosa*^61^. The increase in virulence in the mutant cannot be explained by its colonization ability, but once *ΔpslE* mutant penetrates the leaf, it shows the same phenotype as the cellulose mutant. This suggests that the Psl-like polysaccharide could act as an additional switch between the epiphytic-pathogenic lifestyles with respect to cellulose.

In summary, our work constitutes the report of a Psl-like polysaccharide functioning in a phytopathogenic bacterium, and the obtained results reveal a clear role of this polysaccharide in biofilm formation, plant colonization and virulence, as well as suggest a potential general role during plant-bacteria interactions within the *P. syringae* complex, since the genomic region that encodes Psl is very well conserved. The interconnection observed between the production of the Psl-like polysaccharide and swarming motility suggests a potential correlation between the expression of *psl* and rhamnolipid genes, but further investigation will be required to identify the mechanisms underlying this association.

## Methods

### Bacterial strains and culture conditions

The bacterial strains and plasmids used in this study are listed in Table 1. PssUMAF0158^9^ and mutants were grown in King’s B medium^62^ (KB) supplemented with antibiotics when required and incubated at 25 °C. *Escherichia coli* was used as a host for the mutation and complementation plasmids and was routinely grown on lysogenic broth (LB) at 37 °C. Tryptone-peptone-glycerol (TPG) media^63^ was used for the in vitro experiments. Flow-cell chamber experiments were performed using AB minimal media^64^ supplemented with 0.3 mM glucose and 0.005% yeast extract. The antibiotics used for the selection of PssUMAF0158 mutants were kanamycin 50 mg/L (Km_50_), tetracycline 25 mg/L (Tc_25_), ampicillin 500 mg/L (Ap_500_) and gentamicin 50 mg/L (Gm_50_).

**Table 1:** Bacterial strains and plasmids used in this study.

Strains or plasmids	Relevant characteristics	Reference, source
**Bacterial strain**		
*Pseudomonas syringae* pv. syringae UMAF0158 (PssUMAF0158)	Wild-type, isolated from mango	^28,74^
PssUMAF0158 *Δalg8*	PssUMAF0158 alginate deletional mutant (*alg8* gene); Km^r^	This study
PssUMAF0158 *ΔwssE*	PssUMAF0158 cellulose deletional mutant (*wssE* gene*)*; Km^r^	This study
PssUMAF0158 *ΔpslE*	PssUMAF0158 Psl-like deletional mutant (*pslE* gene); Km^r^	This study
PssUMAF0158 *Δalg8,ΔwssE*	PssUMAF0158 double deletional mutant *alg8*,*wssE*; Km^r^	This study
PssUMAF0158 *Δalg8,ΔpslE*	PssUMAF0158 double deletional mutant *alg8*,*pslE;* Km^r^	This study
PssUMAF0158 *ΔwssE,ΔpslE*	PssUMAF0158 double deletional mutant *wssE*,*pslE;* Km^r^	This study
PssUMAF0158 *Δalg8, ΔwssE,ΔpslE*	PssUMAF0158 triple deletional mutant *alg8, wssE, pslE*; Km^r^	This study
PssUMAF0158 *Δalg8* + pBBR1MCS5*alg8*	Alginate complemented strain with the pBBR1MCS5 plasmid and the *alg8* gene; Gm^r^, Km^r^	This study
PssUMAF0158 *ΔwssE* + pBBR1MCS5*wssE*	Cellulose complemented strain with the pBBR1MCS5 plasmid and the *wssE* gene; Gm^r^, Km^r^	This study
PssUMAF0158 *ΔpslE* + pBBR1MCS5*pslE*	Psl-like complemented strain with the pBBR1MCS5 plasmid and the *pslE* gene; Gm^r^, Km^r^	This study
PssUMAF0158 wild-type GFP	PssUMAF0158 wild-type strain with the pMP4655-GFP plasmid; Tc^r^	This study
PssUMAF0158 wild-type dsRed	PssUMAF0158 wild-type strain with the pMP4662-dsRed plasmid; Tc^r^	This study
PssUMAF0158 *Δalg8* GFP	PssUMAF0158 alginate mutant strain with the pMP4655-GFP plasmid; Km^r^, Tc^r^	This study
PssUMAF0158 *ΔwssE* GFP	PssUMAF0158 cellulose mutant strain with the pMP4655-GFP plasmid; Km^r^, Tc^r^	This study
PssUMAF0158 *ΔwssE* dsRed	PssUMAF0158 cellulose mutant strain with the pMP4655-dsRed plasmid; Km^r^, Tc^r^	This study
PssUMAF0158 *ΔpslE* GFP	PssUMAF0158 Psl-like mutant strain with the pMP4655-GFP plasmid; Km^r^, Tc^r^	This study
PssUMAF0158 *Δalg8,wssE* GFP	PssUMAF0158 *alg8,wssE* double mutant strain with the pMP4655-GFP plasmid; Km^r^, Tc^r^	This study
PssUMAF0158 *Δalg8,pslE* GFP	PssUMAF0158 *alg8,pslE* double mutant strain with the pMP4655-GFP plasmid; Km^r^, Tc^r^	This study
PssUMAF0158 *ΔwssE,pslE* GFP	PssUMAF0158 *wssE,pslE* double mutant strain with the pMP4655-GFP plasmid; Km^r^, Tc^r^	This study
PssUMAF0158 *Δalg8,wssE,pslE* GFP	PssUMAF0158 *alg8,wssE,pslE* triple mutant strain with the pMP4655-GFP plasmid; Km^r^, Tc^r^	This study
PssUMAF0158 *Δalg8* + pBBR1MCS5*alg8* GFP	GFP-tagged alginate complemented strain with the pBBR1MCS5 plasmid and the *alg8* gene; Gm^r^, Km^r^	This study
PssUMAF0158 *ΔwssE* + pBBR1MCS5*wssE* GFP	GFP-tagged cellulose complemented strain with the pBBR1MCS5 plasmid and the *wssE* gene; Gm^r^, Km^r^	This study
PssUMAF0158 *ΔpslE* + pBBR1MCS5*pslE* GFP	GFP-tagged Psl-like complemented strain with the pBBR1MCS5 plasmid and the *pslE* gene; Gm^r^, Km^r^	This study
PssUMAF0158 wt + pBBR1MCS5	PssUMAF0158 wild-type strain with the pBBR1MCS5 plasmid	This study
PssUMAF0158 wt + pBBR1MCS5*pslE*	PssUMAF0158 wild-type strain with the pBBR1MCS5*pslE* complementation plasmid	This study
*E. coli* DH5α	*E. coli [F’ Φ80lacZ ΔM15 Δ(lacZYA-argF)U169 deoR recA endA1 hsdR17 (rK-mK* + *)phoA supE44 lambda- thi-1]*	^75^
*E. coli* mini-Tn7-*kan-gfp*	Donor strain with mini-Tn7-*kan* harbouring *gfp*; Km^r^, Amp^r^	^76^
*E. coli* S17-1 pUX-BF13 (*tnsA-E*)	Helper strain with with a 9.0-kbp EcoRI fragment containing *tnsABCDE*; Amp^r^	^77^
**Plasmid**		
pGEM-T *easy*	3 kb cloning vector, Ap^r^	Promega, Madison, WI
pMP4655	14,2 kb cloning vector harbouring GFP, Tc^r^	^78^
pMP4662	14,2 kb cloning vector harbouring dsRed, Tc^r^	^78^
pGEM-T-KmFRT-HindIII	Contains Km^r^ from pKD4 and HindIII sites, Ap^r^ Km^*r*^	^79^
pFPL2	Contains a flipase gene, Ap^r^	^67^
pBBR1MCS-5	4.7 kb broad-host-range cloning vector, Gm^r^	^80^

### Bioinformatics

Nucleotide and protein sequence searches were performed using the Pseudomonas Genome Database (https://www.pseudomonas.com/) and the National Center for Biotechnology Information (NCBI) database (https://www.ncbi.nlm.nih.gov/). Putative protein domain searches were carried out using Protein Family Software (PFAM) (https://pfam.xfam.org/).

### Strain manipulation and tagging

PssUMAF0158 knockout mutants were constructed using the pGEM-T Easy Vector^®^. First, DNA fragments of approximately 1 kb, corresponding to the 5′ and 3′ flanking regions of the target gene, were amplified and fused using specific primers that included a HindIII site and a T7 primer sequence^65^ (Supplementary Table 3). The resulting product was TA cloned into pGEM-T and fully sequenced to discard any possible mutations. Following sequencing, the resulting plasmid was tagged with the *nptII* Km-resistance gene obtained from pGEM-T-KmFRT-HindIII, yielding pGEM-T-Δgene-Km. For marker-exchange mutagenesis, the pGEM-T-Δgene-Km plasmid was electroporated into PssUMAF0158^66^. Transformants were selected on KB medium containing kanamycin, and the resulting colonies were grown in KB + Km_50_ and KB + Amp_500_ to assess whether each transconjugant exhibited integration of the plasmid into the chromosome or engaged in allelic exchange. Southern blot analyses—using both the gene and Km cassette as probes- were performed to confirm that allelic exchange occurred at a single position and at the correct site within the genome. To generate double and triple mutants, the kanamycin resistance gene was removed using the pFPL2 plasmid^67^. Complemented strains were constructed using specific primers to amplify the selected gene, including fifty base pairs upstream to include the ribosomal binding site. The resulting amplicon was subsequently cloned into the pBBR1MCS5 plasmid and sequenced to discard any possible mutations. Fluorescent tagging of the wild-type and the mutants was performed by electroporation^66^ using the pMP4655 and pMP4662 plasmids. Fluorescent tagging of the complemented strains was first performed by triparental conjugation^68^ using a donor, a helper and each mutant strain. The subsequent introduction of the complementation plasmid by electroporation was performed as mentioned above.

### Plant infection assays

Virulence experiments were carried out^42,69^. Detached tomato leaflets (*Solanum lycopersicum* Mill.) of cv. Hellfrucht Frühstamm were maintained in vitro at 22 °C using Murashige and Skoog medium (MS, Sigma Aldrich). Each leaflet was disinfected, washed, air-dried and inoculated with six 10 μl drops at different points. Inoculations were carried out by piercing with a sterile entomological pin through 10 μl droplets on the leaflet surface. The development of necrotic symptoms was determined after 6 days. For measurement, necrotic areas were digitally analysed using Quantity One 1D Analysis Software. Relative virulence was calculated normalizing the necrotic area values of the tested strains to the wild-type average of each experiment. In parallel, inoculated leaflets were processed for the estimation of the total bacterial population. Tomato leaflets were homogenized in sterile 0.85% NaCl solution, and bacterial counts were determined by plating 10-fold serial dilutions on KB plates with appropriate antibiotics. Four leaflets per strain and experiment and three independent experiments were performed to estimate the induced necrotic area.

### Adhesion assay on mango leaves

The adhesion assays were performed as formerly described^9^ with some modifications. Overnight bacterial cultures were adjusted to 10^8^ CFU/ml. Drops (10 μl) of each strain were inoculated onto 2×2 cm pieces of mango leaves that had been previously disinfected. After 4 h, the leaf pieces were gently washed in 1 ml of sterile 0.85% NaCl solution to remove unattached cells, vigorously vortexed for 30 s in 1 ml of sterile 0.85% NaCl solution to release adhered cells, diluted and plated onto KB plates to determine bacterial numbers. For data normalization, all the cell counts obtained in each experiment were normalized relative to the wild-type average. Two technical replicates per strain and experiment and at least four independent experiments were performed.

### Phylogenetic analysis

The nucleotide sequence of the complete *psl*-like gene cluster, along with partial combined sequences of *rpoD* and *gyrB* housekeeping genes, were used for phylogenetic comparison. To identify the presence of the *psl*-like gene cluster in the genomes of different *P. syringae* strains, the *psl*-like gene cluster of PssUMAF0158 was used for BLASTN comparisons against 34 plant-related strains belonging to different phylogroups within the *Pseudomonas syringae* complex (Supplementary Table 2). Sequence alignment was performed using Clustal Omega^70^, and phylogenetic trees were constructed using MEGA10 software with Jukes Cantor’s algorithm and maximum likelihood (ML) statistical method. The confidence level for the branching points was determined using 1,000 bootstrap replicates. *Pseudomonas aeruginosa* PAO1 was used as an outgroup.

### Congo red assays

Two different approaches were performed. (1) For plate Congo red (CR) binding assays, 10 μl of an overnight culture at 10^8^ CFU/ml (optical density of 0.5 a.u. at 600 nm wavelength) was spotted onto a TPG plate with 20 μg/ml of CR. The samples were incubated at 25 °C for 48 h and images were recorded. Each strain was assayed in triplicate, and three different experiments were performed. (2) For pellicle CR binding, a modified version of the described protocol was performed^34^. Briefly, 100 μl of an overnight culture (10^8^ CFU/ml) was inoculated into 900 μl of TPG medium with 20 μg/ml CR. The samples were incubated 16 h without shaking (static culture) at 25 °C. For the quantification of CR binding, the biofilms were centrifuged at 18,000×*g* to separate the cells (biofilm and non-biofilm formers) from liquid culture, and absorbance of the supernatant of each sample at 490 nm was determined. Free CR exhibits an absorption spectrum from 490 to 530 nm^71^. To calculate CR binding in the pellicle, the supernatant absorbance values of the tested strains were first relativized to those of the control medium with CR for each independent experiment. Then, the relativized data is processed so that the CR binding values of the negative control are cero. Finally, to calculate the fold- change, the obtained values were normalized relative to the wild-type average. Each strain was assayed in triplicate, and three different experiments were performed.

### Biofilm architecture

To assess the differences between the wild-type and mutants regarding biofilm architecture, flow-cell chambers were used^72^. The flow-cell chamber disinfection was carried out for 4 h using a 0.5% hypochlorite solution. Thereafter, the system was washed with sterile distilled water overnight. Biofilms were grown in flow-cells supplied with AB minimal medium supplemented with 0.3 mM glucose and 0.005% yeast extract. Briefly, the flow channels were inoculated with the GFP-tagged wild-type and EPS mutants grown overnight at a low cell density (OD_600nm_ = 0.01 a.u, which corresponds to 10^6^ CFU/ml). The medium flow was kept at a constant rate of 1.3 µl/min by a peristaltic pump (Watson-Marlow 205 S). The incubation temperature was 25 °C. Three independent experiments and at least two technical replicates per experiment and strain were performed. Microscopic inspection and image acquisition were performed using a confocal laser scanning microscope (Leica; DM5500Q) equipped with a 40/1.3 and a 63/1.4 oil objective as well as detector and filter sets for the monitoring of GFP (488 nm for excitation and emission in 501–540 nm). The captured images were analysed with the Leica Application Suite (Mannheim, Germany) and the IMARIS software package (Bitplane, Switzerland) to quantify area and volume values.

### Cellulose staining

Cellulose within the biofilms and bacterial colonies was assayed using calcofluor dye. For this purpose, two different experimental approaches were applied. For biofilm staining, bacteria were grown in flow-cell chambers supplied with AB minimal medium supplemented with 0.3 mM glucose and 0.005% yeast extract. After 12 h of incubation, the flow was stopped, and 300 µl of a 1 mg/ml calcofluor solution was gently injected into the chamber. After 20 min of staining, the flow was restarted, and the unbound dye was cleared for another 20 min. Subsequently, images were obtained, analysed and prepared as indicated above. For the detection of the cells, a 532 nm wavelength was used for the excitation of the dsRed fluorophore, and emission was monitored at 540–730 nm. Calcofluor was excited with a 405 nm wavelength, and emission was monitored at 450–495 nm. For colony staining, 10 μl of an overnight culture (OD_600nm_ = 0.5 a.u, which corresponds to 10^8^ CFU/ml) was spotted onto a TPG plate with 20 μg/ml calcofluor dye. Samples were incubated at 25 °C for 48 h and images of the colonies under UV irradiation were recorded to assess the presence or absence of cellulose. Two independent experiments and at least three technical replicates per experiment and strain were performed.

### Competition experiments

For the competition experiments during biofilm formation, flow-cell chamber experiments were assembled and performed as above described. The chambers were inoculated with a 1:1 mixture of the dsRed-tagged wild-type and GFP-tagged exopolysaccharide mutants, using AB minimal medium supplemented with 0.3 mM glucose and 0.005% yeast extract as carbon sources. Images were recorded, analysed and prepared for publication as indicated in section Biofilm architecture of the Methods. Three independent experiments and at least two technical replicates per experiment and strain were performed.

### Motility assays

For swarming motility analysis, bacteria were stab inoculated in the centre of a 0.5% agar plate with KB medium diluted 20-fold in distilled water. Swarming patterns occur as migrating and branching tendrils from the point of inoculation. After 48 h of incubation at 25 °C, the area of swarming was measured using Quantity One 1D Analysis Software. Three independent experiments and three technical replicates per experiment and strain were performed.

### RNA isolation and quantitative reverse transcription experiments (qRT-PCR)

PssUMAF0158 and respective mutants were grown overnight with shaking in liquid TPG medium at 25 °C. For RNA extractions of biofilms grown on plates, the cultures were adjusted to an OD of 0.5 a.u. at 600 nm (10^8^ CFU/ml) and a 10 µl aliquot was transferred to a TPG plate, which was then incubated at 25 °C. Three colonies were collected, resuspended in a sterile 0.85% NaCl solution and centrifuged at 11,000 × *g* for 2 min. For RNA extractions of biofilms grown on liquid medium, the cultures were adjusted to an OD of 0.5 a.u. at 600 nm (10^8^ CFU/ml) and a 100 µl aliquot was transferred to 900 µl of TPG and incubated without shaking (static culture) at 25 °C. Three pellicles were collected by centrifugation at 11,000×*g* for 2 min. Total RNA was extracted from the pellets using an RNA isolation kit (Macherey-Nagel). The total RNA concentration was determined with a NanoDrop 2000 spectrophotometer (Thermo Fisher Scientific, Waltham, MA, USA), and RNA integrity was assessed by agarose gel electrophoresis. The absence of genomic DNA contamination was checked by PCR amplification of RNA samples using specific primers that amplify the syringomycin B gene (Supplementary Table 3), which is mainly found in the syringae pathovar. Subsequently, DNA-free total RNA was converted to cDNA using Superscript III reverse transcriptase (Invitrogen, Carlsbad, CA, USA) and random primers according to the manufacturer’s instructions. The q-RT-PCR assays were conducted in a CFX384 Touch Real-Time PCR detection system (Bio-Rad, Hercules, CA, USA) using SyBrGreen Supermix (Bio-Rad). The reaction was developed as follows: 2 min at 95 °C (polymerase activation); 1 s at 95 °C; and 5 s at 60 °C. The last two steps were repeated 50 times. Three independent RNA extractions and two technical replicates per extraction were assessed. The expression of *gyrB* and *rpoD* genes were used for normalization of q-RT-PCR data. Gene-specific primers (Supplementary Table 3) were designed using Primer3^73^.

### Reporting summary

Further information on experimental design is available in the Nature Research Reporting Summary linked to this paper.

## Supplementary information

Supplementary Information

Reporting Summary

## Data Availability

The data that support the findings of this study are available from the corresponding author upon reasonable request. The accession numbers of the sequences used in this study have been included in Supplementary Table 2.
