# Supplementary material for: Biological role of EPS from Pseudomonas syringae pv. syringae UMAF0158 extracellular matrix, focusing on a Psl-like polysaccharide
Source: NPJ Biofilms Microbiomes. 2020 Oct 12;6:37. doi: 10.1038/s41522-020-00148-6 (PMC7550585; doi:10.1038/s41522-020-00148-6)
Supplement: Supplementary file 1 — Supplementary Information [file 41522_2020_148_MOESM1_ESM.pdf]

Supplementary Material

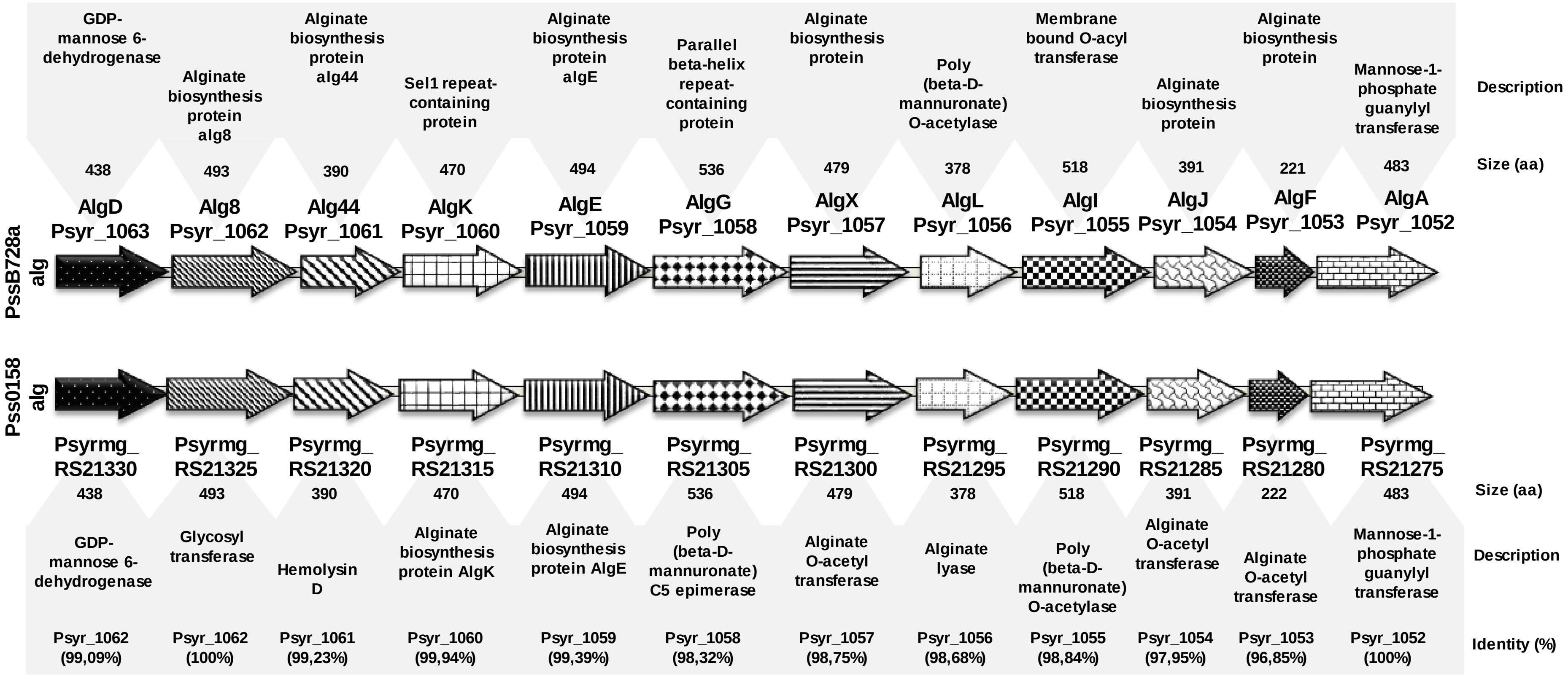

Supplementary Figure 1. Comparison of proteins encoded by the alginate operon of *Pseudomonas syringae* pv. syringae B728a (PssB728a) and *Pseudomonas syringae* pv. syringae UMAF0158 (PssUMAF0158). The Psyr_1052-Psyr_1063 region of PssB728a encodes alginate polysaccharide, and the Psyrmg_RS21275-Psyrmg_RS21330 region of PssUMAF0158 was identified as an orthologue. The figure shows the sizes of the proteins as the number of amino acids, their functions and the percentage of identity between the two

strains being compared. Putative functions have been obtained from the Pseudomonas Genome Database (https://www.pseudomonas.com/).

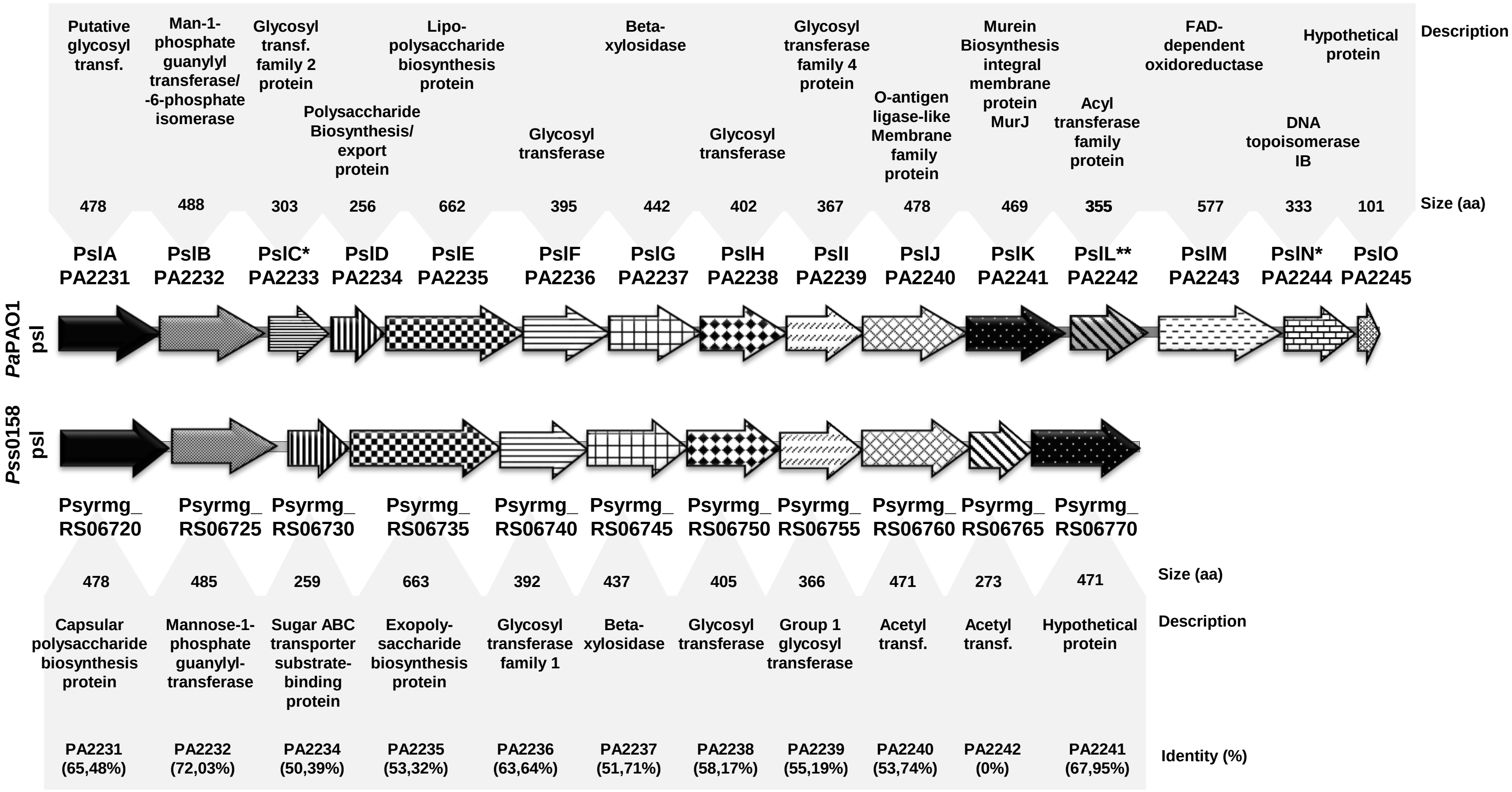

Supplementary Figure 2. Comparison of proteins encoded by the *psl* operon of *P. aeruginosa* PAO1 (PAO1) and PssUMAF0158 (PssUMAF0158). The PA2231-PA2245 region of PAO1 encodes a Psl polysaccharide, and the Psyrmg_RS06720-Psyrmg_RS06770 region of PssUMAF0158 was identified as similar. The PslC-like and PslN-like proteins (*) seem to be encoded outside the *psl*-like cluster at Psyrmg_RS00890 and Psyrmg_RS04445. The *pslM* and *pslO* genes are missing, although they are not required to produce the polysaccharide[29]. The *pslL* gene of PAO1 (**) encodes for an acyltransferase that has no identity with any protein encoded in the

PssUMAF0158 genome. However, the *psyrmg_RS06765* gene, located within the *psl*-like cluster, encodes an acetyltransferase that may replace the role of PslL in PssUMAF0158. In fact, this has already been illustrated in the *P. syringae* B728a strain, in which the acetyltransferase Psyr_3310 encoded by a gene located between *pslJ* and *pslK* may fulfil the normal function of *pslL* in *P. aeruginosa* strains[40]. The figure shows the sizes of the proteins as the number of amino acids, their functions and the percentage of identity between the two strains being compared. Putative functions have been obtained from the Pseudomonas Genome Database (https://www.pseudomonas.com/).

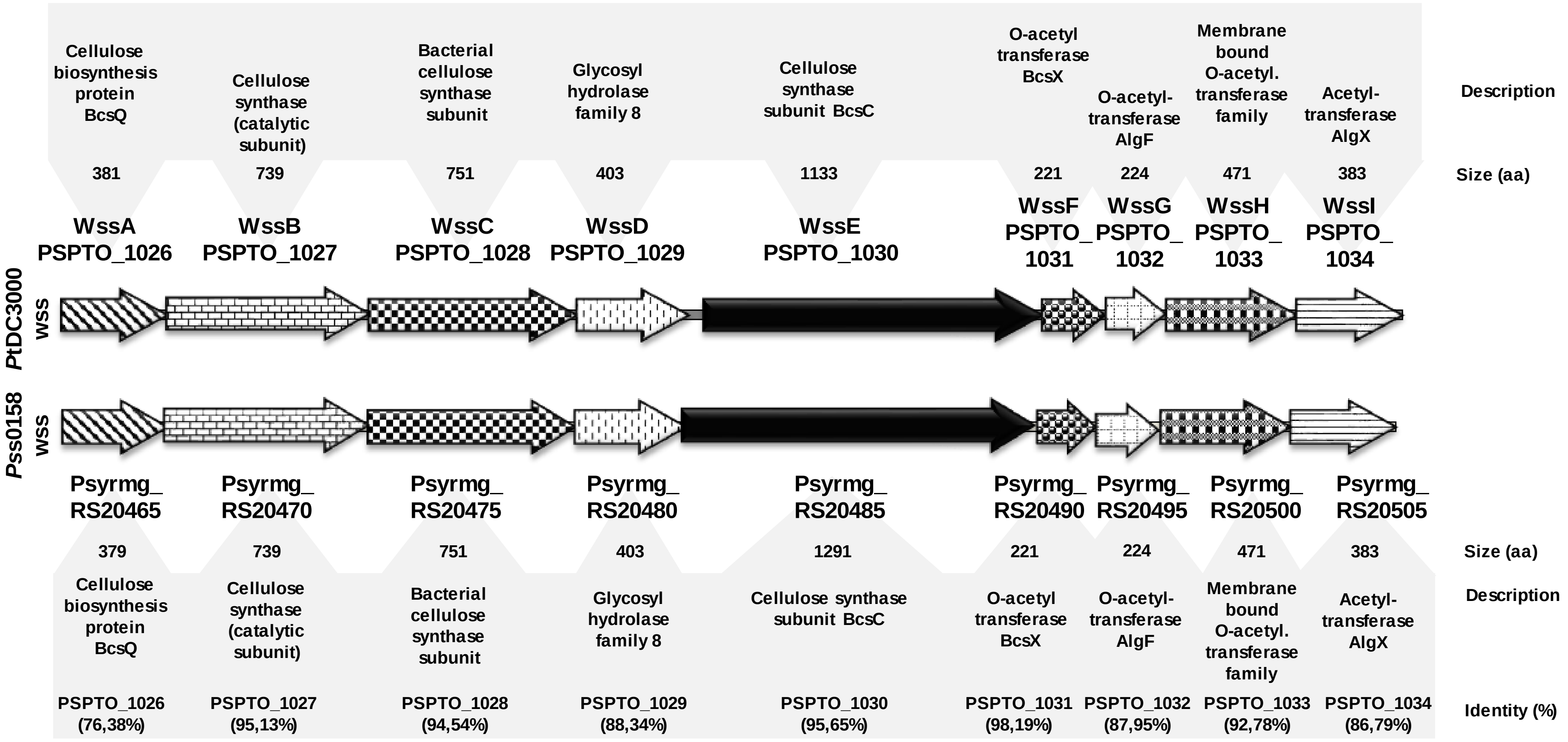

Supplementary Figure 3. Comparison of proteins encoded by the cellulose operon of *P. syringae* pv. tomato DC3000 (PtDC3000) and *P. syringae* pv. syringae UMAF0158 (PssUMAF0158). The Pspto_1026-Pspto_1034 region of PtDC3000 encodes cellulose polysaccharide, and the Psyrmg_RS20465-Psyrmg_RS20505 region of PssUMAF0158 was identified as an orthologue. The figure shows the sizes of the proteins as the number of amino acids, their functions and the percentage of identity between the two strains being

compared. Putative functions have been obtained from the Pseudomonas Genome Database (https://www.pseudomonas.com/) and the Protein Family Software (PFAM) (https://pfam.xfam.org/).

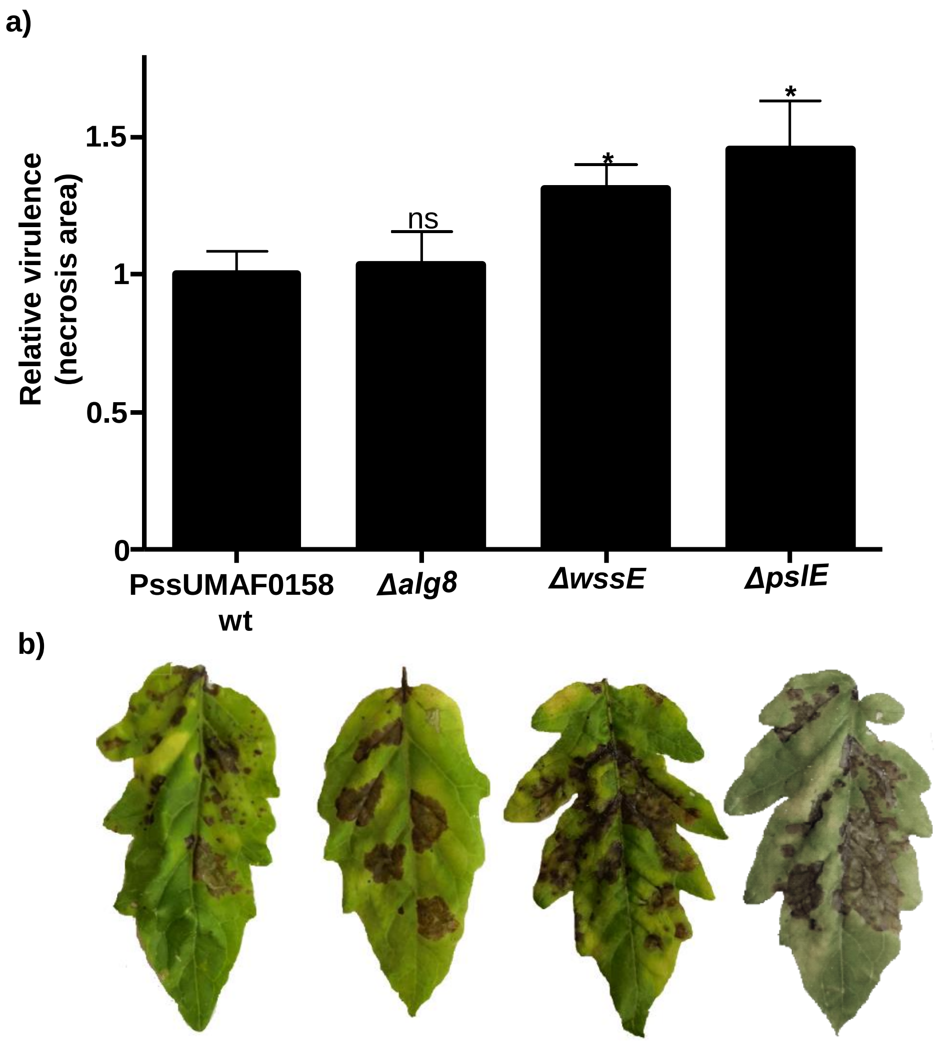

Supplementary Figure 4. Analysis of the alginate, cellulose and Psl-like polysaccharides as putative virulence factors of *P. syringae* pv. syringae UMAF0158. a) Relative virulence of PssUMAF0158 wild-type and extracellular matrix mutants in tomato leaflets measured

by lesion size. Four leaflets per experiment and three independent experiments were performed; b) Representative symptoms developed on tomato leaflets at 6 days postinoculation. No significant differences were found in virulence between wild-type and *Δalg8* mutant. PssUMAF0158 wild-type (PssUMAF0158 wt), PssUMAF0158 alginate mutant (*Δalg8*), PssUMAF0158 cellulose mutant (*ΔwssE*) and PssUMAF0158 Psl-like polysaccharide mutant (*ΔpslE*) were tested. Statistical significance was assessed by two-tailed Mann–Whitney test (* $p<0.05$). Error bars correspond to the standard error (SE).

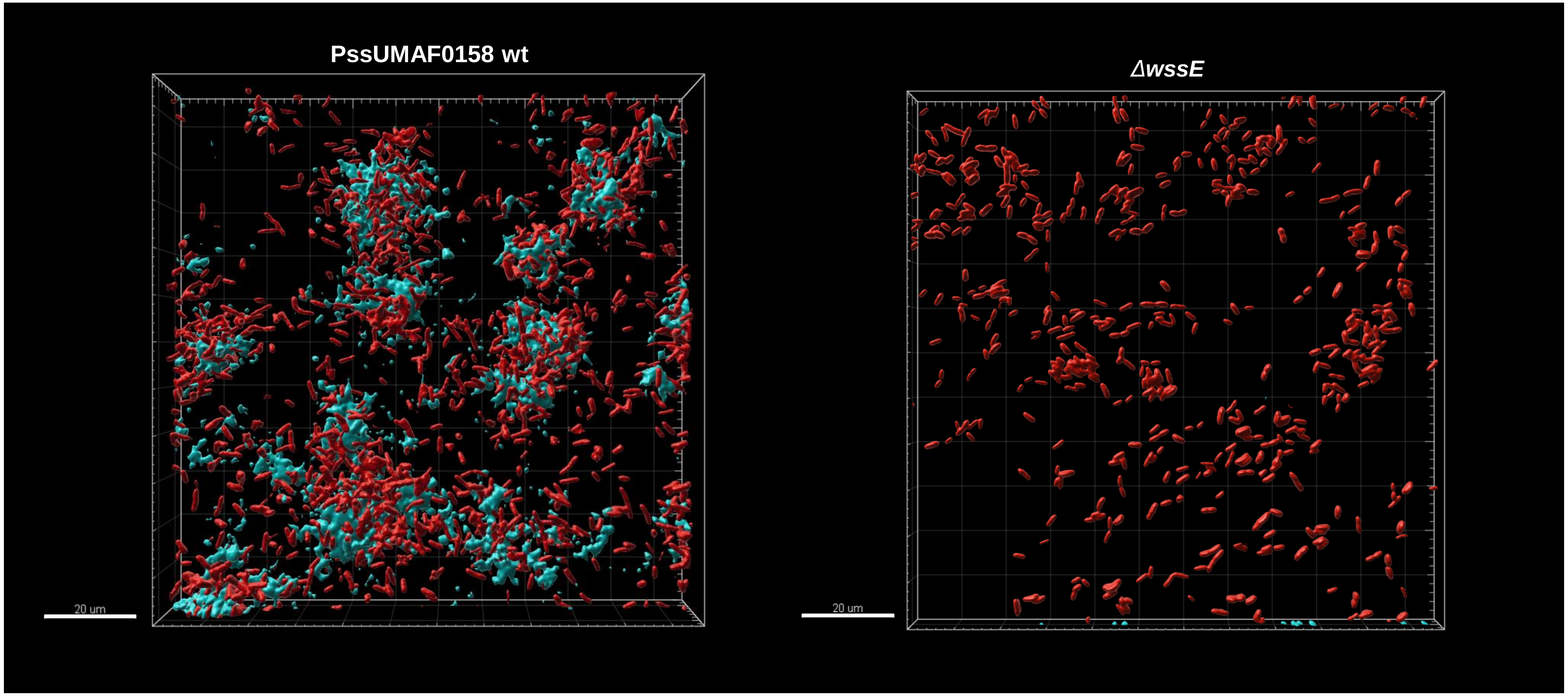

Supplementary Figure 5. Cellulose staining of the biofilm. Representative 12h 3D biofilm images of the dsRed-tagged *P. syringae* pv. syringae UMAF0158 wild-type (PssUMAF0158 wt) and dsRed-tagged cellulose mutant (*ΔwssE)* stained with calcofluor dye (blue) are shown. The obtained images were analysed with the Leica Application Suite (Mannheim, Germany) and the IMARIS software package (Bitplane, Switzerland). Scale bar 20 µm.

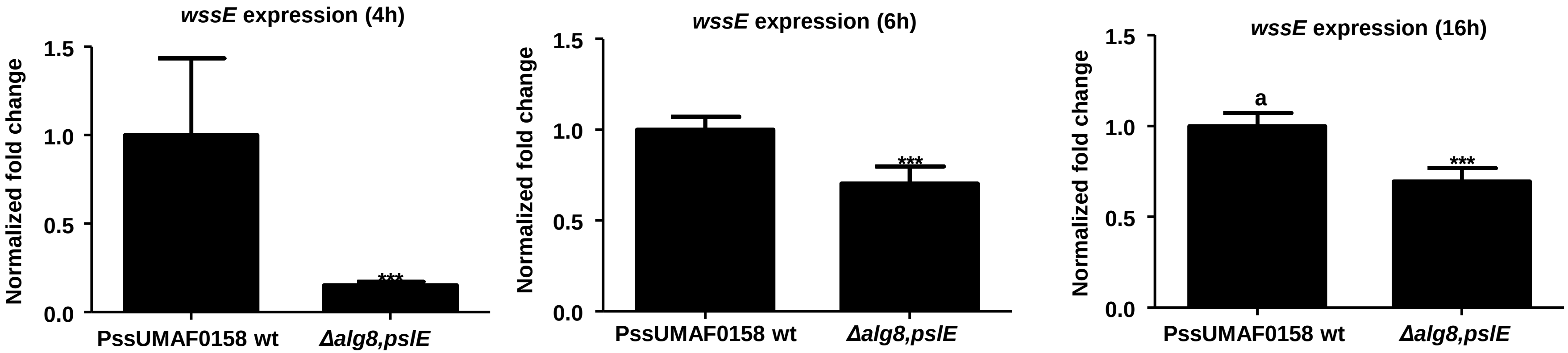

Supplementary Figure 6. qRT-PCR experiments of the *wssE* gene from pellicles after 4, 6- and 16h post-inoculation at 25ºC. Results are shown as the fold-change expression of *wssE* gene in *Δalg8,pslE* double mutant compared to the wild-type. Expression of *wssE* gene is lower in the double mutant compared to the wild-type, which is not consistent with the phenotype observed in *Δalg8,pslE* regarding the binding of Congo red in the pellicles. PssUMAF0158 wild-type (PssUMAF0158 wt) and PssUMAF0158 *Δalg8,pslE* double mutant (*Δalg8,pslE*) were tested. Statistical significance was assessed by two-tailed Mann–Whitney test (***p<0.001). Error bars correspond to the standard error (SE).

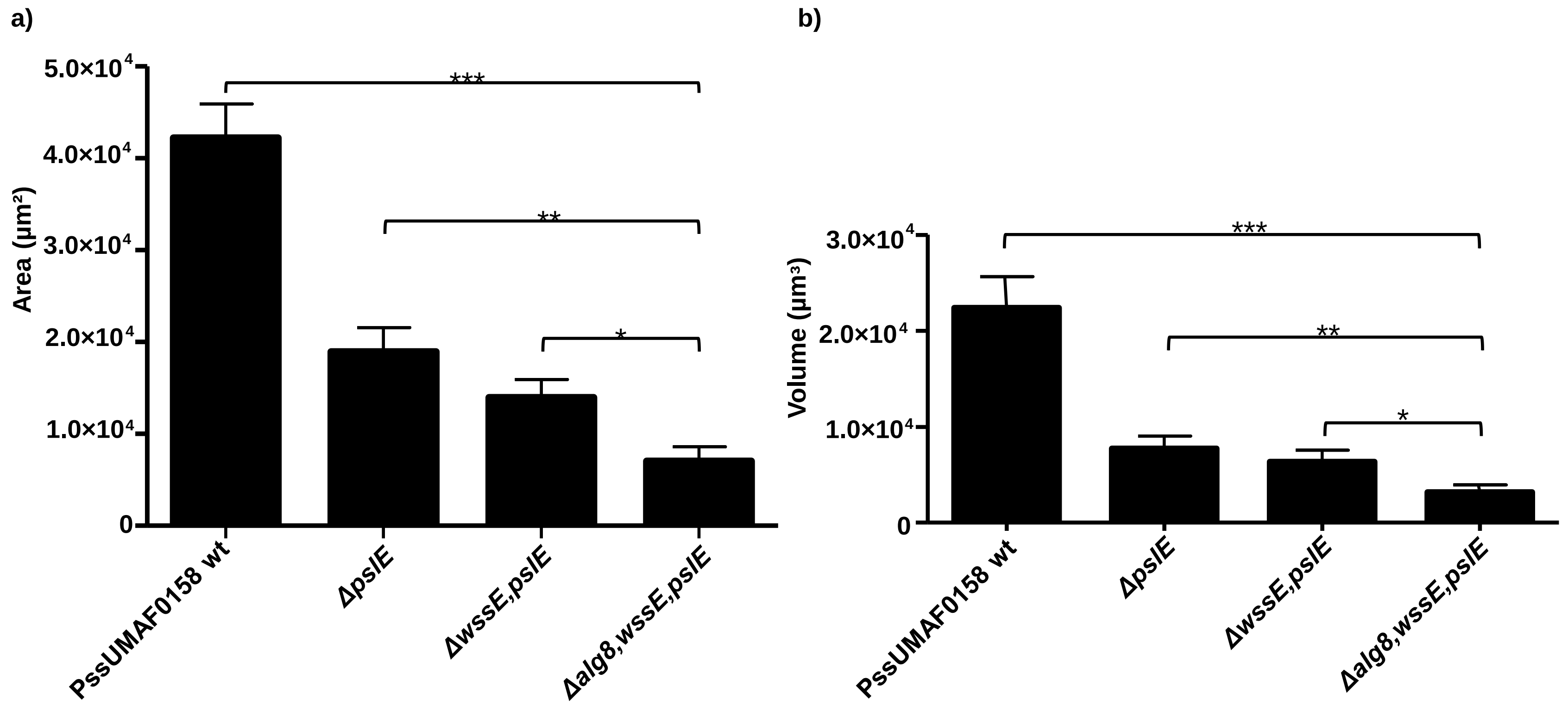

Supplementary Figure 7. Flow-cell chamber experiments. a) Area covered (µm²) by 48h biofilms; b) Volume covered (µm³) by 48h biofilms. PssUMAF0158 wild-type (PssUMAF0158 wt), PssUMAF0158 *ΔpslE* single mutant (*ΔpslE*), PssUMAF0158 *ΔwssE,pslE* double mutant (*ΔwssE,pslE*) and PssUMAF0158 *Δalg8,wssE,pslE* triple mutant (*Δalg8,wssE,pslE*) were tested. Statistical significance was assessed by two-tailed Mann–Whitney test (*p<0.05, **p<0.01, ***p<0.001). Error bars correspond to the standard error (SE).

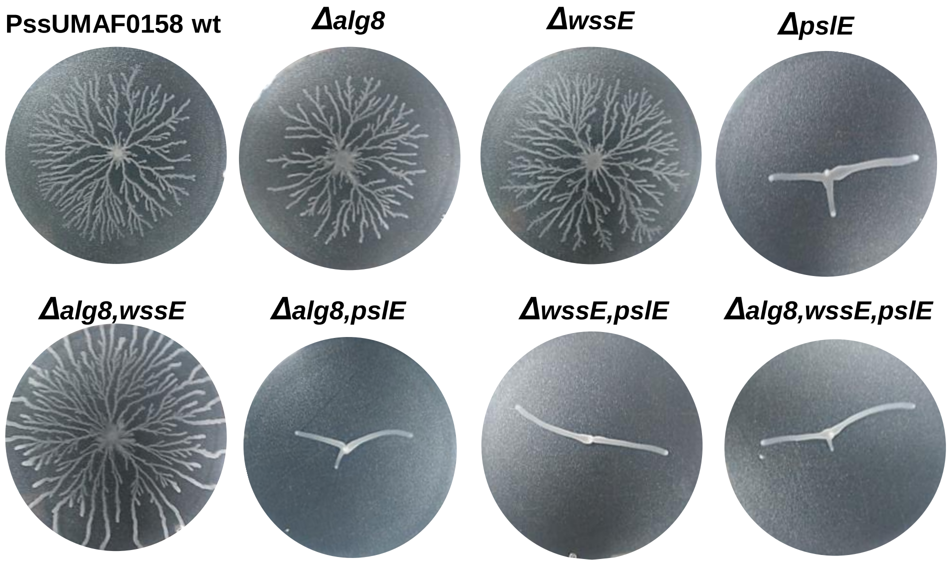

Supplementary Figure 8. Swarming motility. Representative images of swarming plates after 48h of growth at 25ºC. The PssUMAF0158 wild-type (PssUMAF0158 wt), PssUMAF0158 alginate mutant (*Δalg8*), PssUMAF0158 cellulose mutant (*ΔwssE*), PssUMAF0158 Psl-like polysaccharide mutant (*ΔpslE*), PssUMAF0158 *Δalg8,wssE* double mutant (*Δalg8,wssE*), PssUMAF0158 *Δalg8,pslE* double mutant (*Δalg8,pslE*), PssUMAF0158 *ΔwssE,ΔpslE* double mutant (*ΔwssE,pslE*) and PssUMAF0158 *Δalg8,wssE,pslE* triple mutant (*Δalg8,wssE,pslE*) mutants were tested.

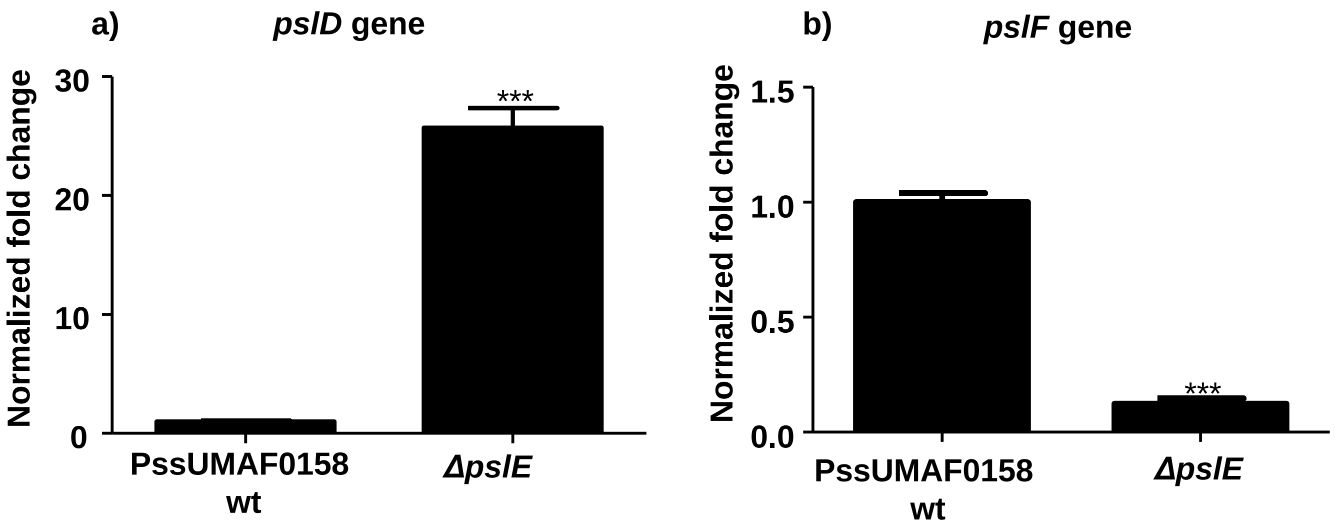

Supplementary Figure 9. qRT-PCR experiments of the *pslD* and *pslF* genes from TPG plates after 48h of growth at 25ºC. Results are shown as the fold-change expression of the selected genes in the *ΔpslE* mutant strain compared to the wild-type. PssUMAF0158 wild-type (PssUMAF0158 wt) and PssUMAF0158 Psl-like mutant (*ΔpslE*) strains were tested. Three independent RNA extractions and two technical replicates per extraction were assessed. Statistical analysis was assessed by two-tailed Mann-Whitney test (***>0.001). Error bars correspond to the standard error (SE).

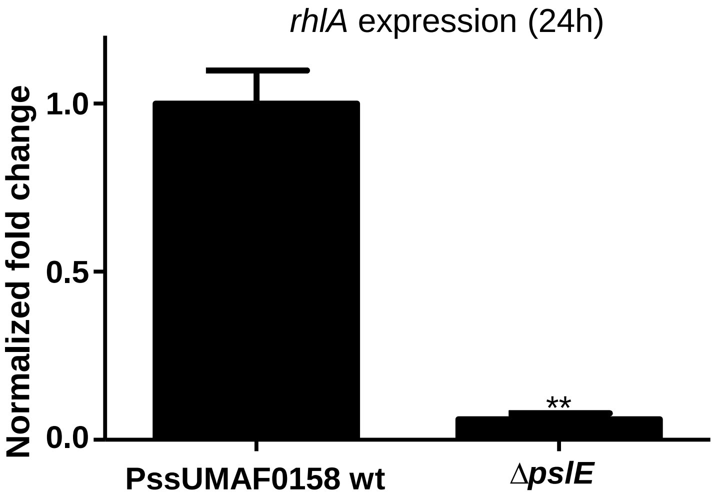

Supplementary Figure 10. qRT-PCR experiments of the *rhlA* gene from TPG plate after 24h of growth at 25ºC. Results are shown as the fold-change expression of *rhlA* gene in *ΔpslE* mutant compared to the wild-type. The expression of *rhlA* gene is lower in the mutant compared to the wild-type, which is consistent with the impairment in swarming motility observed in *ΔpslE* mutant. PssUMAF0158 wild-type (PssUMAF0158 wt) and PssUMAF0158 *ΔpslE* mutant (*ΔpslE*) were tested. Statistical significance was assessed by two-tailed Mann–Whitney test (**$p<0.01$). Error bars correspond to the standard error (SE).

Supplementary Table 1. Domains of proteins encoded by the *psl* cluster of *Pseudomonas aeruginosa* PAO1 and *Pseudomonas syringae* pv. syringae UMAF0158 strains.

Supplementary Table 1a. *Pseudomonas aeruginosa* PAO1 strain.

Protein	Size (aa)	Domains	Domain position	Description
**PslA**	478	CoA_binding_3	70-248	CoA_binding
		Bac_transf	284-472	Bacteria sugar transferase
**PslB**	488	NTP_transferase	9-296	Nucleotidyl transferase
		MannoseP_isomer	325-475	Mannose-6-phosphate isomerase
**PslC**	303	Glyco_transf_2_3	3-220	Glycosyltransferase like family 2
**PslD**	256	Poly_export	49-129	Polysaccharide biosynthesis/export protein
**PslE**	662	GNVR	403-479	G-rich domain on putative tyrosine kinase
**PslF**	395	Glycos_transf_1	192-365	Glycosyl transferases group 1
**PslG**	442	Cellulase	48-285	Glycosyl hydrolase family 5
**PslH**	402	Glyco_trans_4_4	16-206	Glycosyl transferase 4-like domain
		Glyco_trans_1_4	222-359	Glycosyl transferases group 1
**PslI**	367	Glyco_transf_4	17-183	Glycosyltransferase Family 4
		Glyco_trans_1_4	204-308	Glycosyl transferases group 1

**PslJ**	478	-		-
**PslK**	469	MurJ	30-442	Lipid II flippase MurJ
**PslL**	355	Acyl_transf_3	7-322	Acyltransferase family
**PslM**	577	FAD_binding_2	14-549	FAD binding domain
**PslN**	333	Topoisom_I	94-266	Eukaryotic DNA topoisomerase I, catalytic core
**PslO**	101	-	No predicted domains	-

Supplementary Table 1b. ***Pseudomonas syringae*** **pv. syringae UMAF0158 strain.**

Protein	Description	Identity	Size (aa)	Domains	Domain position	Domains description
**PSYRMG_ RS06720**	Capsular polysaccharide biosynthesis protein	65,48% with PslA	478	CoA_binding_3	70-248	CoA_binding
				Bac_transf	284-472	Bacteria sugar transferase
**PSYRMG_ RS06725**	Mannose-1-phosphate guanylyltransferase	72,03% with PslB	485	NTP_transferase	6-294	Nucleotidyl transferase
				MannoseP_isomer	323-473	Mannose-6-phosphate isomerase
***PSYRMG_ RS00890**	Rhamnosyltransferase	48,84% with PslC	308	Glyco_transf_2_3	9-173	Glycosyltransferase like family 2
**PSYRMG_ RS06730**	Sugar ABC transporter substrate-binding protein	50,39% with PslD	259	Poly_export	47-130	Polysaccharide biosynthesis/export protein
**PSYRMG_ RS06735**	Exopolysaccharide biosynthesis protein	53,32% with PslE	663	No predicted domains	-	-
**PSYRMG_ RS06740**	Glycosyl transferase family 1	63,64% with PslF	392	Glycos_transf_1	189-367	Glycosyl transferases group 1
**PSYRMG_ RS06745**	Beta-xylosidase	51,71% with PslG	437	Cellulase	47-279	Glycosyl hydrolase family 5

**PSYRMG_ RS06750**	Glycosyl transferase	58,17% with PslH	405	Glyco_trans_4_4	16-205	Glycosyl transferase 4-like domain
				Glyco_trans_1_4	222-235	Glycosyl transferases group 1
**PSYRMG_ RS06755**	Group 1 glycosyl transferase	55,19% with PslI	366	Glycos_transf_1	200-308	Glycosyl transferases group 1
**PSYRMG_ RS06760**	Hypothetical protein	53,74% with PslJ	471	Wzy_C	252-380	O-Antigen ligase
**PSYRMG_ RS06765**	Acetyltransferase	0%	273	Hexapep	202-235	Bacterial transferase hexapeptide (six repeats)
**PSYRMG_ RS06770**	Hypothetical protein	67,95% with PslK	471	MurJ	28-434	Lipid II flippase MurJ
***PSYRMG_ RS04445**	DNA topoisomerase	59,28% with PslN	356	Topoisom_I	94-292	Eukaryotic DNA topoisomerase I, catalytic core

*These genes are encoded outside the putative *psl*-like cluster.

Supplementary Table 2. Strains belonging to representative plant-associated phylogenetic groups of the *Pseudomonas syringae* complex.

Strain	Code	Isolation	Phylogroup (PG)	Accession number
Pseudomonas syringae pv. aceris strain ICMP2802	PsaICMP2802PG2	Acer sp.	2	NZ_LJPM00000000.1
Pseudomonas syringae pv. aceris strain A10853	PsaA10853PG2	-	2	NZ_LGAR00000000.1
Pseudomonas syringae pv. syringae B728a	PssB728aPG2	Bean	2	NZ_QJTV00000000.1
Pseudomonas syringae pv. syringae B301D	PssB301DPG2	Pear	2	NZ_CP005969.1
Pseudomonas syringae pv. aptata str. DSM50252	PsaDSM50252PG2	Sugar beet	2	AEAN00000000.1
Pseudomonas syringae pv. atrofaciens strain LMG5095	PsaLMG5095PG2	Common wheat	2	NZ_CP028490.1
Pseudomonas syringae pv. syringae UMAF0158	PssUMAF0158PG2	Mango	2	NZ_CP005970.1
Pseudomonas caricapapayae ICMP2855	PcICMP2855PG6	*Carica papaya*	6	NZ_LJPW00000000.1
Pseudomonas syringae pv. helianthi strain ICMP4531	PshICMP4531PG6	Sunflower	6	NZ_LJQM00000000.1
Pseudomonas syringae pv. tagetis strain ICMP4091	PstICMP4091PG6	*Tagetes erecta*	6	NZ_LJRM00000000.1
Pseudomonas savastanoi pv. savastanoi NCPPB 3335	PssNCPPB3335PG3	Olive	6	NZ_CP008742.1
Pseudomonas savastanoi pv. phaseolicola 1448A	Psp1448APG3	Bean	3	NC_005773.3
Pseudomonas savastanoi pv. glycinea strain ICMP2189	PsgICMP2189PG3	Soybean	3	NZ_LJQL00000000.1

Pseudomonas coronafaciens pv. coronafaciens strain 3113	Pc3113PG4	Oat	4	NZ_RBUJ00000000.1
Pseudomonas coronafaciens pv. porri strain ICMP8961	PcpICMP8961PG4	Leek	4	NZ_LJRA00000000.1
Pseudomonas coronafaciens pv. oryzae strain I_6	Pc1_6PG4	Rice	4	NZ_CP046035.1
Pseudomonas coronafaciens pv. garcae strain ICMP4323	PcgICMP4323PG4	Coffee	4	NZ_LJQK00000000.1
Pseudomonas cannabina strain ICMP 2823	PcICMP2823PG5	Hemp	5	NZ_FNKU00000000.1
Pseudomonas cannabina strain ICMP 2821	PcICMP2821PG5	Hemp	5	NZ_RBOW00000000.1
Pseudomonas syringae pv. coriandricola strain ICMP12471	PscICMP12471PG5	Coriander	5	NZ_LJPZ00000000.1
Pseudomonas syringae CC1557	PsCC1557	Snow	10	
Pseudomonas syringae pv. actinidiae str. NCPPB 3871	PsaNCPPB3871PG1	Kiwi	1	NZ_LKEN00000000.1
Pseudomonas syringae pv. actinidiae str. NCPPB 3739	PsaNCPPB3739PG1	Kiwi	1	NZ_AFTH00000000.1
Pseudomonas syringae pv. delphinii strain ICMP529	PsdICMP529PG1	*Delphinium* sp.	1	NZ_LJQH00000000.1
Pseudomonas syringae pv. tomato str. DC3000	Pst3000PG1	Tomato	1	NC_004578.1
Pseudomonas syringae pv. apii strain ICMP2814	PsaICMP2814PG1	Celery	1	NZ_LJPR00000000.1
Pseudomonas syringae pv. tomato NCPPB 1108	PstNCPPB1108PG1	Tomato	1	NZ_ADGA00000000.1
Pseudomonas syringae pv. maculicola strain ICMP3935	PsmICMP3935PG1	Broccoli	1	NZ_LJQR00000000.1

Pseudomonas viridiflava strain CFBP 1590	PvCFBP1590PG7_8	Tomato	7	NZ_LT855380.1
Pseudomonas syringae pv. primulae strain ICMP3956	PspICMP3956PG7	*Primula* sp.	7	NZ_LJRC00000000.1
Pseudomonas syringae pv. ribicola strain ICMP3882	PsrICMP3882PG7	Golden currant	7	NZ_LJRF00000000.1
Pseudomonas cichorii strain ICMP 3353	PcICMP3353PG11	Tomato	11	NZ_RBRE00000000.1
Pseudomonas cichorii strain ICMP 6917	PcICMP6917PG11	Safflower	11	NZ_RBRY00000000.1
Pseudomonas cichorii strain ICMP 1649	PcICMP1649PG11	Celery	11	NZ_RBPN00000000.1
Pseudomonas aeruginosa PAO1	PaPAO1	Outgroup		NC_002516.2

Supplementary Table 3. Primers used in this study.

Primers code	Primers sequence 5'→3'	Use	Reference
*alg8*_fw_up	CGTCGTTATCCCGATTCTGG	To amplify *alg8* upstream sequence (1039 pb)	This study
*alg8*_HindIII_rv_up	*CCCTATAGTGAGTC*AAGCTTCGTATCCCTAAGTCAGTTGC		
*alg8*_HindIII_fw_down	AAGCTT*GACTCACTATAGGG*ATGAATACAGCCGTGAATGC	To amplify *alg8* downstream sequence (1044 pb)	This study
*alg8*_rv_down	CGCTCAGGTTGGTGCTGCTG		
*wssE*_fw_up	CAACCACAGCACCTCCGAAG	To amplify *wssE* upstream sequence (1022 pb)	This study
*wssE*_HindIII_rv_up	*CCCTATAGTGAGTC*AAGCTTGACGTCTCCCAGGATAATTTG		
*wssE*_HindIII_fw_down	AAGCTT*GACTCACTATAGGG*ATGCCTTCCGTTCTTGCCGG	To amplify *wssE* downstream sequence (1023 pb)	This study
*wssE*_rv_down	CTACCGTCGGACTGTGCGGT		
*pslE*_fw_up	CACACGCATCGTCTGGTCAA	To amplify *pslE* upstream sequence (1090 pb)	This study
*pslE*_HindIII_rv_up	*CCCTATAGTGAGTC*AAGCTTGTCTGTTTCCCTGACAATTA		
*pslE*_HindIII_fw_down	AAGCTT*GACTCACTATAGGG*CGGATCGGGAGCCGAGGCTG	To amplify *pslE* downstream sequence (1042 pb)	This study
*pslE*_rv_down	CATGCCGTTGCCATGCGAGA		
Compl_HindIII_*alg8*_fw	AAAAAGCTTGCTTGCAGCCTGGGGCTTGA	To amplify the *alg8* gene and rbs sequence (1530 pb)	This study
Compl_BamHI_*alg8*_rv	AATCTAGATCAAACCATCGTCAACAGCA		

Compl_HindIII_*wssE*_fw<br>Compl_BamHI_*wssE*_rv	AAAAAGCTTCTATCAGTTCCGTGAAGACG<br>AATCTAGATCAGTTGGAATAAGGTGAAC	To amplify the *wssE* gene and rbs sequence (3924 pb)	This study
Compl_HindIII_*pslE*_fw<br>Compl_BamHI_*pslE*_rv	AAAAAGCTTCATGAATTACGAGCTGCGCA<br>AATCTAGATCAGCCTCGGCTCCCGATCC	To amplify the *pslE* gene and rbs sequence (2040 pb)	This study
qRT-PCR158_*gyrB*_F<br>qRT-PCR158_*gyrB*_R	TGCTGACCTTCTTCTTCCGT<br>AGATACCTGGAGCCGATTCG	To amplify a fragment of the *gyrB* gene by q-RT-PCR (198 pb)	This study
qRT-PCR158_*rpoD*_F<br>qRT-PCR158_*rpoD*_R	CGAAGAAGGCATCCGTGAAG<br>CTCAGAACATCGGAAAGGCG	To amplify a fragment of the *rpoD* gene by q-RT-PCR (120 pb)	This study
qRT-PCR158_*rhlA*_F<br>qRT-PCR158_*rhlA*_R	TCAGCCAGATTCGCCAACTA<br>CCTTGCTTTCCAGGTCCAGA	To amplify a fragment of the *rhlA* gene by q-RT-PCR (198 pb)	This study
qPCR_*pslD*_F<br>qPCR_*pslD*_R	TGTTTCAGGTGCTCAACGAC<br>GGGAAATGTTGATGGTCAGC	To amplify a fragment of the *pslD* gene by q-RT-PCR (150 pb)	This study
qPCR_*pslF*_F<br>qPCR_*pslF*_R	CGTGATGGTTCTGCCCTATC<br>GTTGCCATGCGAGACTTCTT	To amplify a fragment of the *pslF* gene by q-RT-	This study

		PCR (154 pb)	
B1	CTTTCCGTGGTCTTGATGAGG	To amplify a fragment of the *syrB* gene (752 pb)	Sorensen et al. 1998
B2	TCGATTTTGCCGTGATGAGTC		

Nucleotide bases in italics show the sequences used for the phusion of the fragments amplified for mutation. Underlined bases show restriction sequences.
